# Structural origins of the Mixed Alkali Effect in Alkali Aluminosilicate Glasses: Molecular Dynamics Study and its Assessment

**DOI:** 10.1038/s41598-020-59875-7

**Published:** 2020-02-19

**Authors:** Federica Lodesani, Maria Cristina Menziani, Hiroyuki Hijiya, Yoichi Takato, Shingo Urata, Alfonso Pedone

**Affiliations:** 1 Department of Chemical and Geological Sciences, University of Modena and Reggio Emilia, via G. Campi 103, 41125 Modena, Italia; 2 Materials Integration Laboratories, AGC Inc., Yokohama, Kanagawa 221-8755 Japan; 3 Innovative Technology Laboratories, AGC Inc., Yokohama, Kanagawa 221-8755 Japan

**Keywords:** Molecular dynamics, Atomistic models

## Abstract

The comprehension of the nonlinear effects provided by mixed alkali effect (MAE) in oxide glasses is useful to optimize glass compositions to achieve specific properties that depend on the mobility of ions, such as the chemical durability, glass transition temperature, viscosity and ionic conductivity. Although molecular dynamics (MD) simulations have already been applied to investigate the MAE on silicates, less effort has been devoted to study such phenomenon in mixed alkali aluminosilicate glasses where alkali cations can act both as modifiers, forming non-bridging oxygens and percolation channels, and as charge compensator of the AlO_4_^−^ units present in the network. Moreover, the ionic conductivity has not been computed yet; thus, the accuracy of the atomistic simulations in reproducing the MAE on the property is still open to question. In this work, we have validated five major interatomic potentials for the classical MD simulations by modelling the structure, density, glass transition temperature and ionic conductivity for three aluminosilicate glasses, (25 − x)Na_2_O − x(K_2_O) − 10(Al_2_O_3_) − 65(SiO_2_) (x = 0, 12.5, 25). It was observed that only the core-shell (CS) polarizable force field well reproduces the experimentally measured MAE on *T*_g_ and the ionic conductivity as well as the higher conductivity of single sodium aluminosilicate glass at low temperature and the higher conductivity of single potassium aluminosilicate glass at high temperature. The MAE is related to the suppression of jump events of the alkaline ions between dissimilar sites in the percolation channels consisting of both sodium and potassium ions as in the case of alkaline silicates. The superior reproducibility of the CS potential is originated from the larger and the flexible ring structures due to the smaller Si-O-Si inter-tetrahedra angle, creating appropriate percolation channels for ion conductivity. We also report detailed assessments for using the potential models including the CS potential for investigating MAE on aluminosilicates.

## Introduction

Ionic conductivity in inorganic glasses is gaining a huge interest for the enormous number of applications that such materials are having in a number of major technological developments in the domains of energy conversion and storage (solid electrolytes in battery and fuel cells) or in the environmental monitoring (solid state ionic membranes for sensors)^1^. Among the various ways to control ionic diffusion and thus ionic conductivity a possibility is that to exploit the so-called Mixed Alkali Effect (MAE) or more in general the Mixed Ion Effect (MIE)^2,3^. This effect refers to a large non-linear deviation in glass properties observed when an alkali cation (or more in general a mobile ion) is gradually replaced by another alkali cation. The ionic conductivity of glasses exhibiting the MAE shows a deep minimum when half of the alkaline ions of type A are replaced by type B ions, meaning that cation mobility progressively reduces up to a substitution ratio equal to unity. The minimum is more pronounced at low temperature where the activation energy for ionic diffusion has a maximum and it usually reduces with increasing the temperature. This phenomenon, which is one of the most intriguing challenges in glass science, is observed also for other dynamical properties such as diffusion, viscosity, chemical durability and the glass transition temperature^4^, whereas the other properties such as molar volume, density, elastic modulus or refractive index demonstrate smaller deviations from linear additivity rules^2^.

The origin of the MAE has been deeply investigated from experiments and several phenomenological and physical models have been proposed during the last decades^5–8^. Among them, the dynamic structure model (DSM)^9^ and the random ion distribution model (RIDM)^10^ are the more accepted ones. Both models assume that cations are randomly dispersed throughout the glass matrix in matching sites that are specific and adapted to allocate each cationic species; the hopping between dissimilar sites is strongly hindered or blocked due to structural or energy mismatch due to the ion size difference. For the DSM model structural relaxation of the sites after occupation by a dissimilar cation can occur below the glass transition temperature, whereas the RIDM model assumes that ion hopping occurs within a static glass structure.

In the last years, several common structural hypotheses, such as the fact that cations are homogeneously mixed and retain their distinct local order as in the single alkali glasses, have been confirmed by neutron and X-ray diffraction experiments^11,12^, NMR measurements^13,14^ as well as by classical MD simulations^15–18^. The latter technique, in particular, has been of great help to elucidate the complex ion dynamics and mechanism of ionic diffusion within percolation channels created by modifier cations and to explain the MAE as a suppression of the mobility of cations since the contemporary presence of two cations of different size block one another’s passage^15–17,19^. It is well known that the accuracy of the MD simulation results in describing the structure and properties of materials (inorganic glasses in this context) strongly depends on the interatomic potential models used to obtain forces between ions for the integration of the equation of motion. In the last decades, a variety of interatomic potentials for the different families of oxide glasses (silicates, aluminosilicates, borosilicate, phosphosilicate etc…) have been developed^20–30^. The majority of them were optimized by fitting on experimental data, whereas the others adopted quantum mechanical data (usually coming from ab initio MD simulations)^26,30^. With few exceptions^20^, since structural data were included in the fitting data, they can generally be safely used to investigate glass structure but the accuracy on calculation of the other properties such as ionic conductibility is open to question.

The main aim of this study is to evaluate the performance of several interatomic potentials available in the literature in reproducing the microstructure, density, glass transition temperature and ionic conductivity (or for more convenience the resistivity ρ that is its inverse) of Na- K- aluminosilicate glasses. The glass compositions studied are (25 − x)Na_2_O − xK_2_O − 10Al_2_O_3_ − 65SiO_2_ (x = 0, 12.5 and 25), named as SAN, SANK and SAK hereafter. The reason for the choice of these glass compositions is that addition of aluminum to a silicate improves chemical durability and decreases viscosity and liquidus temperature, allowing us to develop practically available glass substrates in a wide range of applications. In addition, it has already been found that the sodium-potassium mixed aluminosilicate shows MAE on the thermodynamical properties^31^. We also demonstrate the MAE on the glasses by measuring resistivity at three temperature conditions in this work.

The manuscript is organized as follows: detailed descriptions of the force-fields examined as well as the computational protocols and theoretical background used to compute glass properties are explained in section 2. Experimental procedures are also briefly mentioned in this section. Then, in section 3, after describing all results on mixed alkaline effects found in both MD simulations and the resistivity measurements, relations between glass structures and MAE are deeply discussed. Finally, we summarize important findings as conclusions in section 4.

## Computational and Experimental Methods

### Force-Fields description

Five interatomic potential models were examined and four of them are the rigid ionic types parameterized by Pedone^20,32^, Teter^21^, Kob^26^ and Guillot-Sator^27^ groups. Hereafter, these potential models are abbreviated using the first letters of the authors’ name as PMMCS, Teter, SHIK and GS, respectively. The other one is the core-shell (CS) model with parameters of Si-O, Al-O, Na-O and O-O interactions taken from references^22,33,34^. In this work, a parameter set for the K ion of the CS model has been developed since it was absent in the literature. The functional forms and parameters are described in the followings.

#### PMMCS potentials

The original version of the PMMCS potential combines a long-range Coulomb potential and a short-range Morse function with a repulsive contribution of the form *B*_*ij*_*/r*^12^, in order to prevent atomic collapse at high temperature and pressure:

1 $${U}_{ij}({r}_{ij})=\frac{{z}_{i}{z}_{j}{e}^{2}}{{r}_{ij}}+{D}_{ij}[{(1-{e}^{-{a}_{ij}({r}_{ij}-{r}_{ij}^{0})})}^{2}-1]+\frac{{B}_{ij}}{{{r}_{ij}}^{12}}$$

where *D*_*ij*_, *a*_*ij*_ and ${r}_{ij}^{0}$ are the parameters for the *i*-*j* pairs of the Morse function and *z*_*i*_ are the partial charges of ions *i*, which are described as rigid cores; the partial charges on the cations are referred to the fixed charge of −1.2e assigned to the oxygen. The PMMCS potential was parameterized to reproduce the experimental crystal structures and properties such as elastic constants. However, since the original parameters for the Al-O interaction overestimates the amount of Three-Bridging-Oxygens (TBO) and 5-fold coordinated Al as discussed in ref. ^35^, we borrow the Al-O interaction using a Buckingham function with a parameter set of ref. ^21^:

2 $${U}_{ij}({r}_{ij})=\frac{{z}_{i}{z}_{j}{e}^{2}}{{r}_{ij}}+{A}_{ij}{e}^{-{r}_{ij}/{\rho }_{ij}}-\frac{{C}_{ij}}{{{r}_{ij}}^{6}}+\frac{{B}_{ij}}{{{r}_{ij}}^{12}}$$

The parameter set is summarized in Table S1 of the ESI.

#### Teter and GS potentials

As for the PMMCS potentials, the Teter and GS potentials are based on the rigid ions with partial charges on atoms that interact through pairwise Buckingam potentials using Eq.(2) without the last term. The partial charge of oxygen (*z*_O_) is −0.945 and −1.2 for GS and Teter potentials, respectively, and all of the parameters, *A*_*ij*_, *r*_*ij*_ and *C*_*ij*_, are summarized in Tables S2 and S3 of the ESI. To avoid the Buckingham catastrophe at short distances, a repulsive term of the form *D*_*ij*_/*r*^6^ and a splice correction of the form $\frac{{B}_{ij}}{{r}_{ij}^{n}}+{D}_{ij}\cdot {r}_{ij}^{2}$ are applied at small *r* for the GS and Teter potentials, respectively. Both potentials were parameterized through an empirical fitting on structures of silicate crystals and amorphous systems.

#### SHIK potentials

In this potential, the long-range electrostatic interactions are evaluated by means of the Wolf truncation method^36^, whereas the Buckingham functional form is used for the short range interactions:

3 $${U}_{ij}({r}_{ij})={U}_{ij}^{W}({r}_{ij})+{A}_{ij}{e}^{-\frac{{r}_{ij}}{{\rho }_{ij}}}-\frac{{C}_{ij}}{{{r}_{ij}}^{6}}+\frac{{D}_{ij}}{{r}_{ij}^{24}}$$

where

4 $${U}_{ij}^{W}({r}_{ij})={z}_{i}{z}_{j}[\frac{1}{{r}_{ij}}-\frac{1}{{r}_{cut}^{W}}+\frac{({r}_{ij}-{r}_{cut}^{W})}{{({r}_{cut}^{W})}^{2}}]$$

The Coulombic interactions are truncated at ${r}_{cut}^{W}$ = 10 Å, whereas the van der Waals interactions are truncated at a distance of 8 Å. The Si, Al, K and Na charges are fixed, whereas the oxygen charge depends on the glass composition as follows^26^:

5 $${z}_{O}=\frac{(1-{y}_{X}-{y}_{Al}){z}_{Si}+2{y}_{X}{z}_{X}+2{y}_{Al}{z}_{Al}}{{y}_{X}-{y}_{Al}-2}\,\,where\,\,X=Na,K$$

*y*_*α*_ and *z*_*α*_ are the mole fractions of the oxides and charges of the species α, respectively. The parameters, *A*_*ij*_, *r*_*ij*_, *C*_*ij*_ and *D*_*ij*_, have been determined to reproduce the pair distribution functions evaluated by ab initio MD simulations of melts of silicate^37^ and aluminosilicate^26^ systems at high temperature. (see in Table S4 of the ESI).

#### Core-Shell (CS) model potentials

In this model, the total charge *Z* of the oxygen ions is split into a core with charge *Z*+*Y* and a massless shell with charge −*Y*, and they are coupled by a harmonic spring with force constant *k*_*s*_^38^. Besides the damped harmonic interaction with the corresponding core, the oxygen shells interact with each other and with Si, Al, K and Na cations through a short range Buckingham term, whereas electrostatic forces act among all species, which bear full formal charges. In addition, the three-body screened harmonic potentials are used to control the intra-tetrahedral O-Si-O and O-Al-O angles. The full expression of the potential is given by:

6 $${U}_{ij}({r}_{ij},{r}_{c-s},{\theta }_{ijk})=\frac{{z}_{i}{z}_{j}{e}^{2}}{{r}_{ij}^{2}}+{A}_{ij}{e}^{-(\frac{{r}_{ij}}{{\rho }_{ij}})}-\frac{{C}_{ij}}{{r}_{ij}^{6}}+\frac{1}{2}{k}_{s}{({r}_{c-s})}^{2}+\frac{1}{2}{k}_{b}{({\theta }_{ijk}-{\theta }_{ijk}^{0})}^{2}{e}^{-(\frac{{r}_{ij}}{\rho }+\frac{{r}_{jk}}{\rho })}$$

The employed parameters^22,34^ are reported in Table S5 of the ESI. It is worth to highlight that the K-O interatomic potential parameters have been fitted in this work using the relaxed method^39^ implemented in the GULP code^40^ on the structure of K_2_Si_2_O_5_, K_6_Si_2_O_7_ and KAlSiO_4_ taken from the MINCRYST database^41^. In this method, the potential parameters are varied in order to minimize the difference between the experimental structure (lattice parameters and atomic positions) and the one optimized at the molecular mechanics level of theory.

The lattice parameters and bond distances computed with the new potentials are compared with the experimental data as shown in Table S6 of the ESI. The maximum discrepancies of 3% have been found, confirming that the parameter set is well optimized.

### Generation of glass structure

Glass structural models containing 5120 atoms were generated through the melt and quench approach by MD simulations^32^. Four replicas of each glass model have been examined to confirm the reproducibility of the results and to estimate the variability in the glass properties. The leap-frog algorithm encoded in the DL_POLY2.14 package^42^ was used to integrate the equations of motion with a time step of 0.2 fs and 2 fs for the core-shell potential and the other potentials, respectively. The initial configurations were generated by randomly placing the atoms in a cubic box, whose size corresponds to the experimental density. Table 1 lists the number of the atomic species and the experimental density used to determine the unit cell size.

**Table 1:** Atomic composition of each simulation box and relative experimental density.

*Glass*		Al	Si	Na	K	O	TOT	Density (g/cm^3^)
X = 0	SAN	320	1040	800	0	2960	5120	2.4711
X = 12.5	SANK	320	1040	400	400	2960	5120	2.4702
X = 25	SAK	320	1040	0	800	2960	5120	2.4478

The quenching was carried out using both NVT and NPT ensembles. The latter has been used to determine the glass transition temperature. The systems were heated and hold at 3500 K (in cases of PMMCS, Teter, GS, SHIK potentials) or 3200 K (in the case of CS) for 100 ps, a time required to ensure a sufficient melting of the samples. The liquids were then monotonically cooled to 300 K with a cooling rate of ~5 K/ps. The resulting glass structures were subjected to a final equilibration run of 200 ps. In all cases, the temperature and pressure (0.1 MPa) were controlled using the Berendsen thermostat and barostats^43^ with frictional constants set to 0.2 ps. The glass transition temperature was determined as a crossing temperature, where two extrapolated linear lines of the specific volume at lower and higher temperature regions. This is schematically shown in Figure S1 of the ESI.

The coulomb interactions were calculated by the Ewald summation method except for the SHIK potential. The cutoff distance is 12 Å for the PMMCS, Teter and CS potentials, 11 Å for the GS potential and 10 Å for the SHIK potential. The short-range interactions were evaluated using cutoff values of 8.0 Å for Teter and SHIK potentials, 11 Å for GS potential, 7.5 Å for the CS potential and 5.5 Å for the PMMCS potential.

### Ionic conductivity calculation

The total conductivity (*σ*) is calculated as the sum of contributions (*σ*_*i*_) provided by each ionic mobile species (*i*) in the system:

7 $$\sigma =\sum _{i}{\sigma }_{i}=\sum _{i}{c}_{i}e|{z}_{i}|{\mu }_{i}$$

where *c*_*i*_ is the concentration of the charge carrier *i*, *z*_*i*_ is the charge and *μ*_*i*_ is the ionic mobility, which is defined as the average drift velocity per electric field and is computed in this work through the Nernst-Einstein relation

8 $${\mu }_{i}=\frac{e|{z}_{i}|{D}_{i}}{{k}_{B}T}$$

where *k*_*B*_ is the Boltzmann constant and *D*_*i*_ is the diffusion coefficient that can be computed through the Einstein relation:

9 $$D=\frac{{r}^{2}}{2{n}_{d}\Delta t}$$

where *n*_*d*_ is the dimensionality of the simulation system, Δ*t* is the change in time over which diffusion is calculated and *r*^2^ is the Mean Square Displacement (MSD) expressed as

10 $${r}^{2}=\langle \Delta \overrightarrow{x}{(t)}^{2}\rangle =\frac{1}{N}{\sum }_{i=1}^{N}{({\overrightarrow{x}}_{i}(t)-{\overrightarrow{x}}_{i}(0))}^{2}$$

where *N* is the number of averaged particles, ${\overrightarrow{x}}_{i}(0)\,$is an initial reference position, ${\overrightarrow{x}}_{i}(t)$ is the position at time *t*. To compute the ionic conductivity, NPT-MD simulations of 10 ns were performed for the quenched systems exposed to an electric field of 5 × 10^5^ V/m at temperature below the glass transition temperature of each system. It is important to note that an effective concentration of charge carriers was presumed. That means that only the alkali ions that make at least a jump during the simulation timeframe were considered in Eq. (7). Note that we increased the time step to 0.5 fs for the conductivity calculations with CS model since the CS potential is computationally expensive. A few tests confirmed that the mean square displacement is not influenced by the new timestep.

To study the diffusion mechanism of the alkali cations, the self (*G*_*s*_(*r*, *t*)) and distinct (*G*_*d*_(*r*, *t*)) parts of the van Hove correlation function have been also computed. The self-correlation function is defined as follows:

11 $${G}_{s}(\overrightarrow{r},t)=\frac{1}{N}{\sum }_{i=1}^{N}\delta \langle {\overrightarrow{r}}_{i}(t)-{\overrightarrow{r}}_{i}(0)-\overrightarrow{r}\rangle $$

where $\overrightarrow{r}$ is the travel of an ion in a time *t* and thus it gives information on the number of jumps of the ions. Contrarily, the distinct part of the van Hove function, which can be defined for Na-Na, K-K, Na-K and K-Na pairs, is defined as:

12 $${G}_{d}^{\alpha ,\beta }(\overrightarrow{r},t)=\frac{1}{{N}_{\alpha }}{\sum }_{i=1}^{{N}_{\alpha }}{\sum }_{j=1}^{{N}_{\beta }}\langle \delta (\overrightarrow{r}-{\overrightarrow{r}}_{i}^{\alpha }(0)+{\overrightarrow{r}}_{j}^{\beta }(t))\rangle $$

where *N*_*α*_ and *N*_*β*_ are the number of particles of species α and β and the self-term *i* = *j* is not considered if *α* = *β*.

### Experimental measurements

The glass samples were prepared using SiO_2_ (MORIMURA, 99.5%), Al_2_O_3_ (Kanto Chemical, 99.0%), Na_2_CO_3_ (Kanto Chemical, 99.8%) and K_2_CO_3_ (Kanto Chemical, 99.5%) reagents. After mixing the ingredients, it was melted in a platinum crucible at 1823 K for an hour at air condition using a heating rate of 10 K/min. The sample was annealed at temperature higher than the glass transition temperature in 50 K for an hour, followed by a cooling to room temperature with a quenching rate of 1 K/min.

The density of the obtained glass sample was measured by Archimedes method and the glass transition temperature was determined by measuring the thermal expansion using a thermo-mechanical analyzer (TMA; TD5000SA). For the electrical resistivity measurement, an aluminum deposited glass sample with 50 × 50 × 4 mm size was prepared, then the resistance was measured at 323 to 473 K. According to the resistance-temperature dependence, the resistivity was determined by assuming the Arrhenius equation.

## Results and Discussion

In this session, we firstly examine the five interatomic potentials to investigate their ability for reproducing the mixed alkaline effect on the glass transition temperature and ion conductivity. Then, we compare the short and middle range structures of the glass models to unravel the intrinsic origins of the mixed alkaline effect.

### Glass transition temperature

The estimations of the glass transition temperature (*T*_g_) and density of multicomponent oxide glasses are of paramount importance for industrial applications. However, studies using MD simulations to evaluate *T*_g_ of oxide glasses are very limited so far, even though *T*_g_ of polymers have been quantitatively estimated by MD simulations since 90′s^44,45^. The possible reasons are: (1) most of the studies have applied MD simulations to get insights into the details of the amorphous glassy structures, which are difficult to be elucidated using experimental techniques solely, (2) it is well known that both *T*_g_ and density strongly depend on the quenching rate, and thus the models should have higher fictive temperature and lower density in computational simulations compared to experimental ones. (3) The huge overestimation of *T*_g_ is also due to the application of the periodic boundary conditions (PBCs) in the MD simulations. This is because the simulated system is, in fact, an infinite solid without any surfaces from which the melting would be initiated in the real materials of finite size. Due to the absence of the surfaces, the computational models are susceptible to superheating and supercooling, thereby an effective temperature in MD simulations is usually higher than experimental one. The variation may be ascribed to differences between thermodynamic melting and mechanical melting processes that occur in the case of MD simulations with periodic boundary conditions^46^.

**Figure 1:**
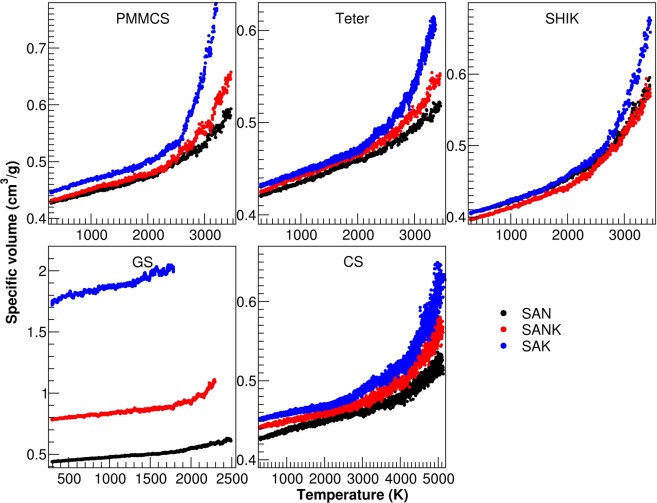
Specific volume against temperature of the three glasses studied with the five interatomic potentials.

Therefore, we only focus on the qualitative assessment of the five potential models for the trends experimentally measured. Figure 1 compares temperature-specific volume (T-V) curves calculated with NPT ensemble for the three glass compositions with different interatomic potential models. Here we only draw the result of one of the four replica simulations. Note that we could not obtain reasonable T-V curves for the GS potential because the models are very unstable at high temperature when potassium is present although we tried several cooling procedures by varying the starting temperature, barostat algorithms, timesteps as well as velocity scaling intervals. The CS model is also very unstable under NPT conditions at high temperature, and this is probably the reason why only NVT simulations have been reported so far with this potential. To avoid the difficulty, a few considerations have to be done for the CS potential. Firstly, an equilibrated model at low temperature (300 K) was slowly melted up using a heating ramp of about 5 K/ps, otherwise the simulation box suddenly expands. Secondly, we found that the velocity-scaling interval is the most important tunable parameter. Indeed, during the quenching without velocity scaling, the temperature of the shell dramatically increases and it escapes from its core. On the contrary, if the velocity scaling is applied at every timestep, the simulation is more stable; however, ionic mobilities increase too much because the kinetic energy is redistributed homogenously on all the system particles equally. As a result, the simulation box also significantly expands, and it is not recovered at room temperature, giving very low densities eventually. After several tests, we reached the best results, in terms of stability and avoiding volume expansion, by scaling the velocities every 100 MD steps. The last point regards the mass of the shell. As mentioned above, the shell can have a very high velocity because of its lightweight. Thus, the mass chosen for these simulations is 0.3 uma to maintain lower velocity and less probability for the shell to lose the core. Thanks to the modified procedures, we were able to heat the systems up to 5000 K to evaluate *T*_g_ of the CS models.

The density and *T*_g_ values determined from the simulations are reported in Table 2, together with the experimental values. The SHIK potential gives the least error on density (≤2%) for all glasses, but it does not reproduce the experimental trend as a function of potassium content correctly. The other potentials give the right trend with a relatively larger error (Teter ≤5%, PMMCS and CS ≤ 10%).

**Table 2:** Experimental and calculated data values of density and glass transition temperature for the SAN, SANK and SAK glasses simulated with the different interatomic potentials (PMMCS, Teter, SHIK and CS).

		SAN		SANK		SAK	
*Density (g/cm*^*3*^*)*	EXP	2.4711	**ERR%**	2.4702	**ERR%**	2.4478	**ERR%**
	PMMCS	2.3297 (0.0114)	5.72%	2.3056 (0.0152)	6.66%	2.2020 (0.0334)	10.04%
	Teter	2.3751 (0.0080)	3.88%	2.3562 (0.0113)	4.61%	2.3243 (0.0145)	5.05%
	SHIK	2.4779 (0.0119)	0.28%	2.5243 (0.0046)	2.19%	2.4663 (0.0076)	0.76%
	CS	2.3325 (0.0148)	5.61%	2.2758 (0.0140)	7.87%	2.2072 (0.0301)	9.83%
*Tg (K)*	EXP	810 (5)		785 (5)		889 (5)	
	PMMCS	2592 (40)		2781 (39)		2664 (82)	
	Teter	2845 (41)		2701 (66)		2603 (18)	
	SHIK	2714 (25)		2621 (37)		2779 (50)	
	CS	3791 (53)		3717 (93)		3910 (32)	

In terms of *T*_g_, as expected, all the potentials hugely overestimate the experimental values (Table 2). The degree of overestimation is proportional to the charges assigned to the ions in each force field since the electrostatic interactions would dominate the cohesive energies of the systems. In fact, the *T*_g_ simulated by PMMCS, Teter and SHIK models range in the narrow temperature region (around 2600 to 2800 K) due to the similar atomic charges, while higher temperatures (around 3700 to 3900 K) are obtained with the CS potential, which assigns larger charges to the ions. Moreover, the steep slope of the volume change in the liquid state leads to the intrinsic and large error of the approach used to determine the *T*_g_.

It is therefore we put priority on reproducing the MAE trend, which is clearly seen in the experimental data in Table 2, rather than absolute values of the *T*_g_. Indeed, the experimental *T*_g_ follows the typical nonlinear trend of MAE when the mixed alkali effect comes into play and shows a minimum for the SANK glass. Another important feature is that the glass transition temperature of SAK is higher than that of SAN. These trends are nicely reproduced by SHIK and CS potentials, whereas PMMCS shows a maximum for SANK and the *T*_g_ varies as a convex curve with increasing potassium. In the case of Teter potential, *T*_g_ monotonically decreases as a function of potassium content. Therefore, the latter two potentials would not be suitable to investigate the glass transition temperature of aluminosilicates with mixed alkaline ions.

### Ionic conductivity and resistivity

The resistivity experimentally measured for the three glasses at three temperatures (297, 371 and 421 K) are reported in Table 3. The expected MAE trend is obviously observed with a maximum in the resistivity (minimum in ionic conductivity) for the mixed alkali glass SANK. Another important phenomenon is that, at low temperature, the resistivity of the SAK glass is larger than that of the SAN glass, whereas the opposite is observed at higher temperature, demonstrating that the mobility of potassium increases more rapidly than that of sodium with increase of temperature.

**Table 3:** Experimental and calculated logarithm of resistivity, ρ, measured along X direction for the SAN, SANK and SAK glasses simulated with the different interatomic potentials (PMMCS, Teter, SHIK, GS and CS).

log ρ (ρ in cm·Ω) X direction							
EXP	T (K)	SAN	SANK	SAK	ΔA	ΔB	ΔC
	279.4	8.34	13.26	8.82	4.92	4.44	0.48
	371.6	6.20	9.88	6.62	3.68	3.26	0.42
	421.1	6.16	8.29	5.65	2.13	2.64	−0.51
PMMCS	550	3.15 (0.04)	3.18 (0.06)	2.51 (0.09)	0.03	0.67	−0.64
	650	2.58 (0.06)	2.68 (0.10)	2.06 (0.07)	0.09	0.62	−0.52
	800	1.88 (0.01)	1.94 (0.07)	1.56 (0.08)	0.05	0.38	−0.32
	950	1.37 (0.06)	1.47 (0.05)	1.27 (0.03)	0.10	0.20	−0.10
Teter	550	3.05 (0.05)	3.60 (0.10)	3.12 (0.04)	0.55	0.48	0.07
	650	2.44 (0.18)	2.96 (0.06)	2.62 (0.07)	0.52	0.35	0.17
	800	1.83 (0.13)	2.30 (0.03)	2.00 (0.10)	0.46	0.30	0.16
	950	1.35 (0.09)	1.79 (0.05)	1.71 (0.03)	0.44	0.08	0.36
SHIK	650	2.94 (0.14)	4.03 (0.08)	4.43 (0.18)	1.09	−0.40	1.48
	800	2.09 (0.09)	2.93 (0.05)	3.45 (0.14)	0.85	−0.52	1.36
	950	1.59 (0.02)	2.31 (0.05)	2.82 (0.21)	0.72	−0.51	1.24
GS	370	2.64 (0.11)	2.41 (0.09)	1.22 (0.05)	−0.23	1.19	−1.42
	420	2.35 (0.05)	2.06 (0.03)	1.00 (0.02)	−0.28	1.06	−1.34
	500	1.87 (0.08)	1.65 (0.07)	0.77 (0.10)	−0.22	0.89	−1.11
CS	950	2.94 (0.13)	3.74 (0.13)	3.21 (0.04)	0.80	0.53	0.27
	1190	1.85 (0.08)	2.22 (0.01)	1.70 (0.04)	0.38	0.53	−0.15
	1350	1.58 (0.07)	1.84 (0.02)	1.46 (0.03)	0.27	0.39	−0.12

The last three columns: ΔA and ΔB are the differences of log ρ between SANK and SAN or SAK respectively, ΔC is the difference between SAK and SAN. Standard deviations are reported in parenthesis.

The temperatures at which the resistivity were experimentally measured are too low to be presumed in the MD simulations since no jumps (and thus insufficient diffusion) of Na and K cations are observed in the timeframe of the NPT-MD simulations of 10 ns, except for the case of the GS potential, which partially demonstrates sufficient diffusion at 370 K. Therefore, the simulation temperatures were set at 370, 420 and 500 K for GS potential, 550, 650, 800 and 950 K for PMMCS and Teter potentials, 650, 800 and 950 K for SHIK potential and 950, 1190 and 1350 K for the CS one.

In order to evaluate the conductivity of glasses, it is necessary to calculate the diffusion coefficient, which can be obtained from the slope of the mean square displacement against simulation time. As an example, the MSD of the sodium ion in the SAN glass is shown in Figure S2 of the ESI, in which the linear increase of the MSD is observed, revealing that the system is in a diffusion regime. With this assumption, the diffusion coefficient is obtained from the slope of the linear part of the curve, as described in Eq. (10) in the method’s section. According to the diffusion coefficients, the ionic mobilities and conductivities were calculated following the procedure explained in the computational details.

**Figure 2:**
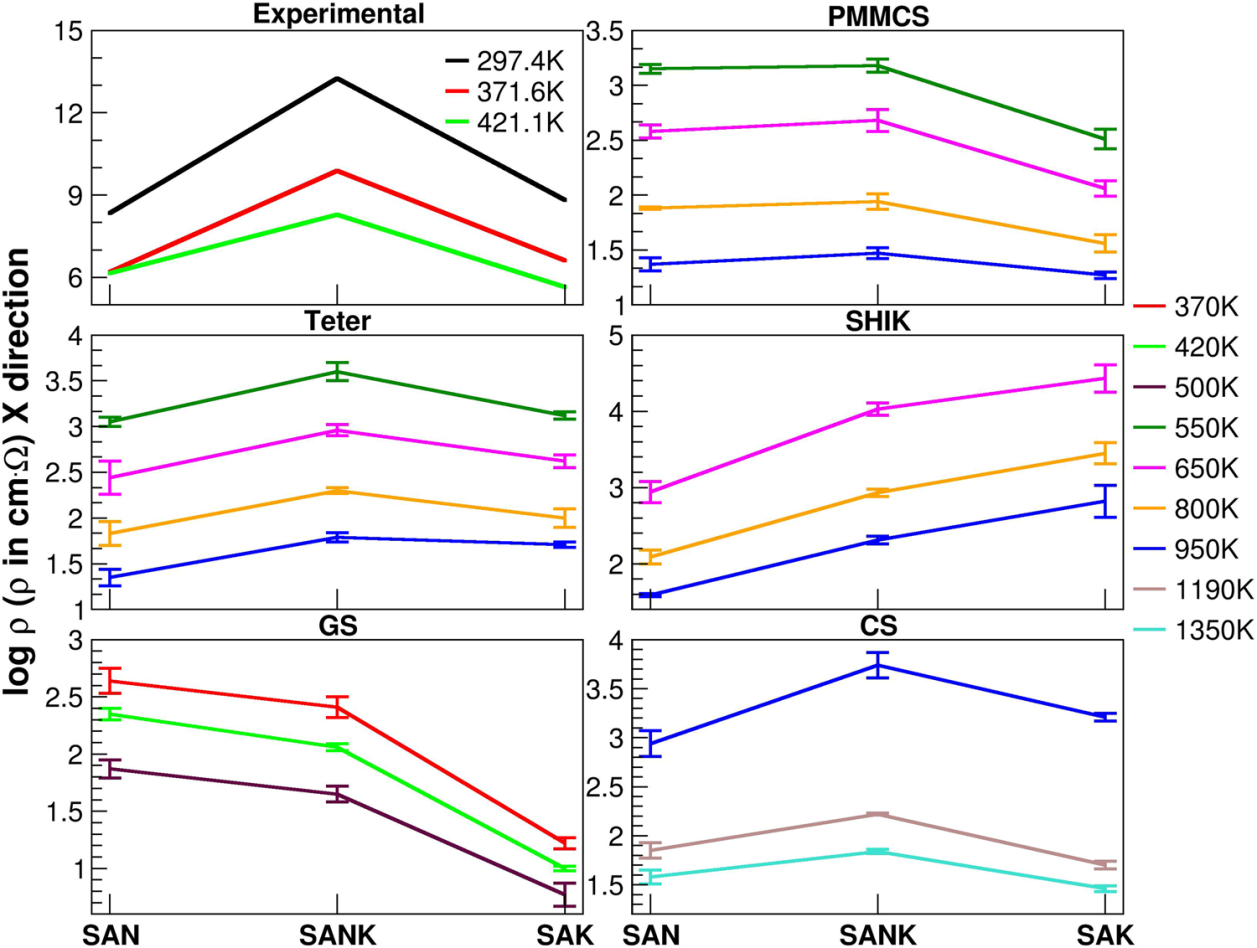
Logarithm of resistivity obtained for the three glasses studied at variable temperature by different interatomic potentials.

A comparison between experimental and calculated resistivity (ρ) of the three glass compositions is reported in Table 3. The last three columns of the table report the differences of log ρ between SANK and SAN (ΔA) or SAK (ΔB), and that between SAK and SAN (ΔC). As in the case of *T*_g_ estimation, because of the intrinsic limitations of the MD simulation and the choice of different temperature conditions, the comparison must be done on trends but not on absolute values of the resistivity. To visually compare the data reported in Table 3, we have plotted the log ρ at different temperatures for the three glasses in Fig. 2. The mixed alkali effect is successfully reproduced by the PMMCS, Teter and CS potentials, whereas the GS and SHIK potentials fail to find MAE and show a monotonic (opposite) trend with potassium addition. The experimental data (Table 3) show that the resistivity of SAN is higher than that of SAK at low temperature, while the SAN possesses lower resistivity than SAK at high temperature. Importantly, the CS model is the only force field, which reasonably reproduces this trend as well as MAE on the resistivity.

To understand the relation between the resistivity and ion mobility, in Table S7 of the ESI, the ionic mobility of Na and K ions in the simulated glasses at the applied temperatures are summarized. As expected, ionic mobility increases with increasing temperature. In general, a Na ion is more mobile than a K ion at all temperatures when the PMMCS, Teter and SHIK potentials are used, whereas, surprisingly, the opposite is observed with the GS potentials. On the contrary, with the CS model Na ions are more mobile at low temperature but the mobility of K ions increases more rapidly with increasing temperature, and consequently, potassium is more mobile than sodium at 1100 and 1350 K.

As discussed later in more detail, the different trend obtained by the five potentials can be rationalized to the variations observed in the average ring size for the three glasses shown in Table 4. When Na ions are replaced by K ions, the average size increases, in the case of the CS model. A similar behavior was recently observed for mixed Na and K silicate glasses^47^.

**Table 4:** Average ring size of the glasses for the different potentials.

Average ring size					
	PMMCS	Teter	SHIK	GS	CS
SAN	8.00	7.92	8.08	7.96	8.03
SANK	7.97	7.86	8.09	7.42	8.31
SAK	8.01	7.95	8.36	6.65	8.54

**Figure 3:**
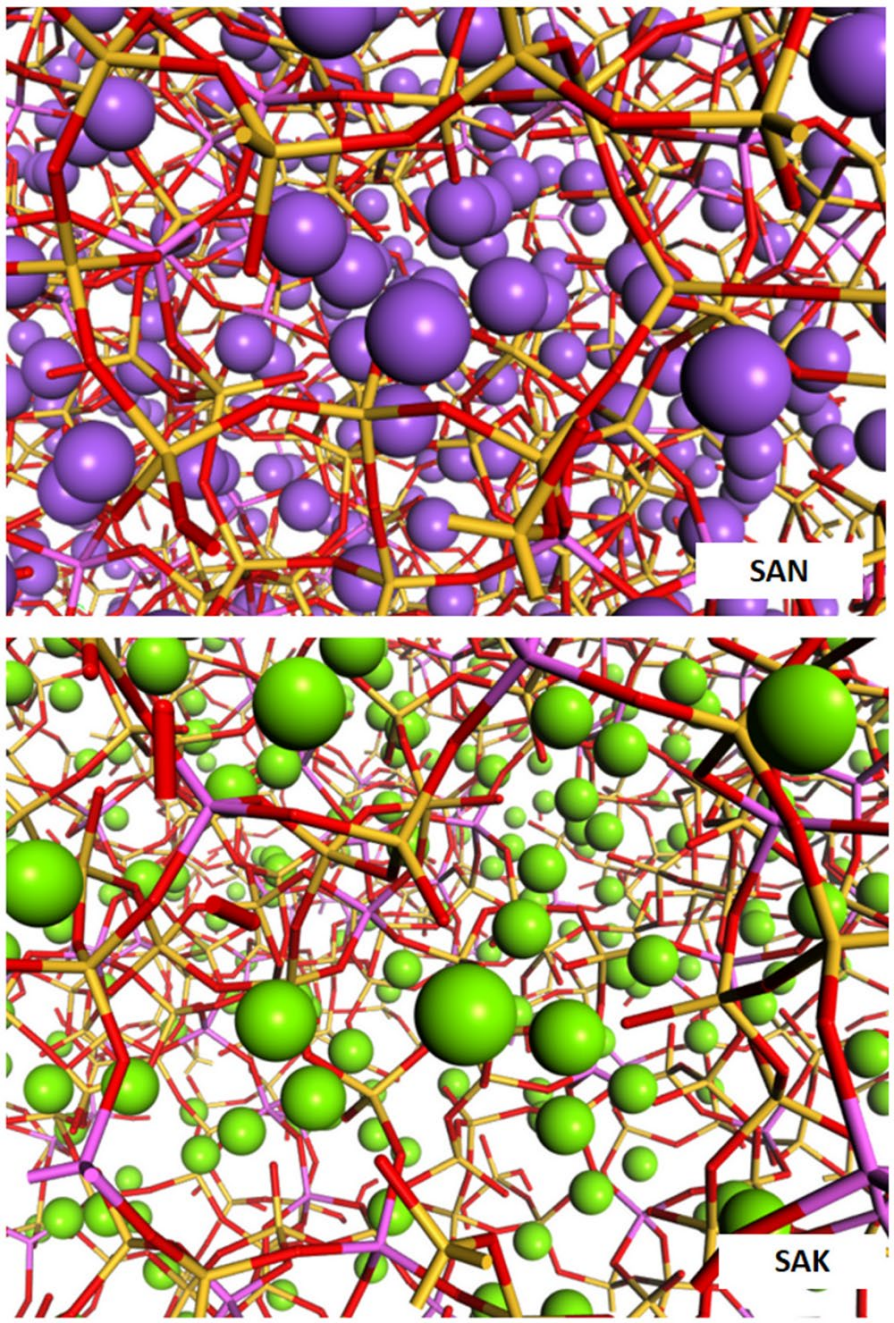
View of typical percolation channels formed in SAN and SAK glasses generated with the CS potential. Silicon and aluminium are represented as yellow and magenta sticks whereas sodium and potassium as cyan and green spheres, respectively.

Since the larger rings are more flexible than smaller ones, the former can expand more when temperature increases. Indeed, the volume expansion with temperature of the SAK glass is greater than those of the SAN and SANK glasses. Consequently, the percolation channels of the alkaline ions are developed in SAK more than in SAN as drawn in Fig. 3. At higher temperature, the more pronounced percolation channels composed of the larger rings in the SANK glass would increase K ion mobility. As a result, at low temperature, a sodium ion diffuses more rapidly than potassium because of its smaller size and weight, but it turns at higher temperature owing to the microstructure developed. Note that this behavior is not observed with the Teter and PMMCS potentials, which provide similar ring size distributions for SAN and SAK glasses, demonstrating that the appropriate middle range structure is crucial for reproducing MAE on the mobility of cations. We would discuss this in the following.

**Figure 4:**
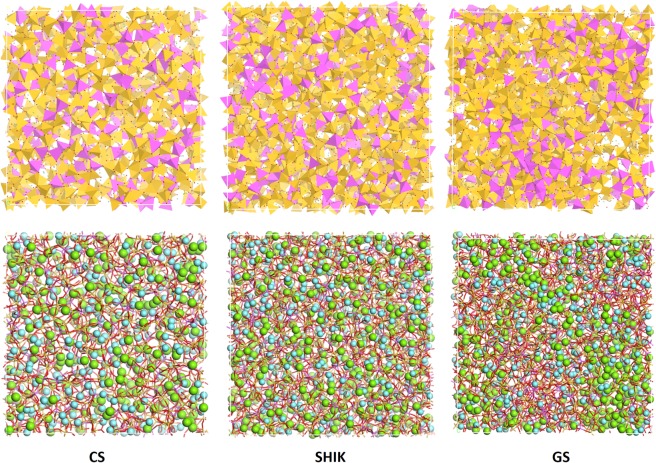
Snapshots of the SANK glass structure generated using the core-shell (CS), SHIK and GS potentials. In the upper panel the silicon (yellow) and aluminium (magenta) ions are represented as polyhedral. In the lower panel, silicon and aluminium are represented as sticks whereas sodium and potassium as cyan and green spheres, respectively.

Table S7 shows that the ionic mobility of Na and K ions in the mixed alkali glass SANK, are lower than those computed for the single alkali glass except for the GS potential, which estimates the larger ionic mobility with more potassium content. This may be due to the formation of three-dimensional K-rich domains as shown in Fig. 4 instead of developing the percolation channels, resulting that the total conductibility is governed by K ion mobility in the model. Note that the SHIK potential shows lower mobility of K ion of one order of magnitude compared with the other potentials. Further, the K ion mobility is less than Na ion, and those in the SANK and SAK glasses are comparable.

**Figure 5:**
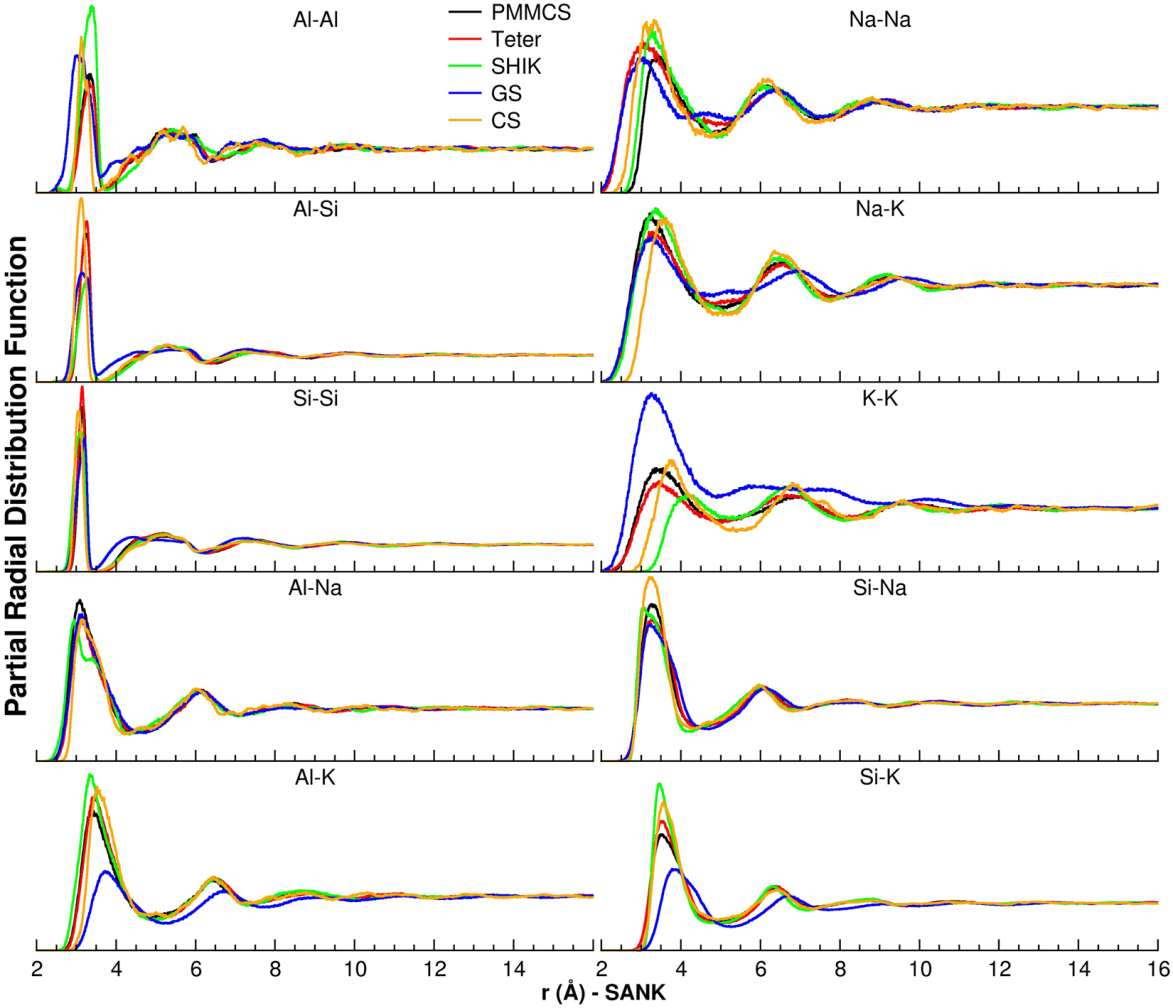
Cation-Cation pair distribution functions of the SANK glass computed using the different interatomic potentials.

These inadequate behaviors are probably due to the strong affinity of potassium ions to the surrounding aluminum ions in the case of SHIK potential with respect to the others, since more intense and narrower first peaks are visually observed in the Al-K pair distribution functions in Fig. 5.

**Figure 6:**
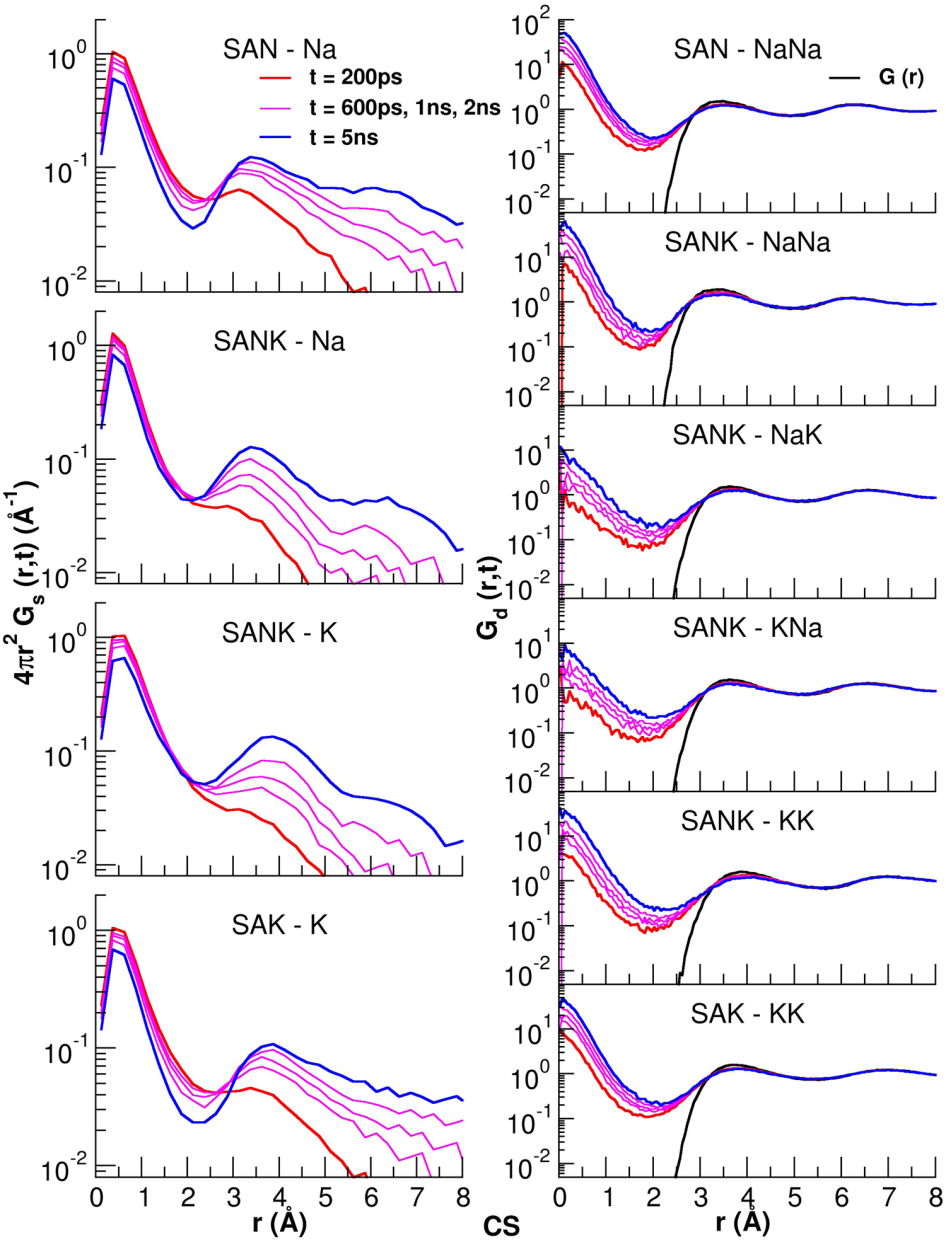
Self (left panels) and distinct (right panels) van Hove correlation functions at different simulation times for alkali ions in the three glasses simulated using the CS potential.

Figure 6 shows the self and distinct part of the van Hove correlation functions computed at 950 K for the three glasses simulated with the CS potential. The correlation functions for the other potentials are reported in Figures S3–S6 of the ESI for comparison. The self-correlation functions for Na and K ions possess the first peak at 0.1 Å, representing localized motion of the ions around the sites initially occupied in short time. The profile slightly decreases with time, then the second and the third peaks at around 3.5 (4.0) Å and 6.5 (7.0) Å, respectively, arise at longer simulation time for sodium (and potassium), representing that both ions make two jumps in about 5 ns of the MD simulations. The profiles of the self-correlation functions for the mixed alkali glass, SANK, are similar to those of the SAN and SAK glasses, but the second and the third peaks developed at longer timescale are clearly suppressed, denoting that the long distance jumps of the ions are restricted in the SANK glass.

The nature of the migration of the alkali ions, Na or K, to a vacancy site, which had been occupied by the identical or the other type of ions, can be studied by analyzing the evolution of the peak close to 0.1 Å as a function of time in the distinct part of the van Hove correlation function. This increment is due to the creation of a vacancy by moving out of an ion and filling the vacancy by another ion jumping into the site. Figure 6 shows that this peak height increases considerably for Na-Na and K-K pairs, reflecting the fact that the high probability of synchronized jumps of two identical ions. Contrarily, the peak height is lower for the mixed alkaline pair of Na-K in the SANK glass, indicating that sharing a site by the different ions is restricted and it would be the intrinsic origin of the MAE. As already reported in the literature studied alkaline silicate glasses^16^, the jumps between dissimilar sites are hampered by energetic and steric constraints and this is the reason of the mixed alkali effect on the transport properties. The previous study investigated Li-K ions mixture, which has larger difference in cation sizes, while, in this study, it was revealed that the CS potential successfully reproduces the same phenomenon in the aluminosilicate glasses with Na-K ions mixing.

### The microstructure

In this section, the three-dimensional structure of the three aluminosilicate glasses, SAN, SANK and SAK, are analyzed to unravel the reasons why only CS potential can reproduce MAE of *T*_g_ and ion conductivity as discussed above. To do so, a variety of structural analyses, such as pair distribution functions, Q^n^ species distribution, percentages and angle distributions of T-O-T bridges (T = Si and/or Al) as well as ring size and cations distributions are investigated as follows.

#### Short-range order

Information on the most probable distances, the average distances and coordination numbers (CN) is obtained by the cation-oxygen pair distribution functions (PDFs). As summarized in the ESI, short range environments of Si-O and Al-O obtained by the five interatomic potentials are very similar and consistent with experimental measurements^42,43^. Even for the partitioning of oxygens between non-bridging oxygens (NBO), bridging oxygens (BO) and three bridging oxygens (TBO), all the potentials produce similar structures with ~16% of NBO and ~83% of BO and less than 0.4% of TBO. The GS model is an exception since it shows a slightly higher amount of NBOs and TBOs (up to 4%) at the expense of BOs as compared in Table 5. Readers can find the cation-NBO/BO/TBO distances and coordination numbers in Table S8 and Figure S7 of the ESI.

**Table 5:** Percentage of the different oxygen typologies (NBO, BO, and TBO) in the SAN, SANK and SAK glasses simulated with the different interatomic potentials (PMMCS, Teter, SHIK, GS and CS).

	%Oxygen	PMMCS	Teter	SHIK	GS	CS
SAN	NBO	16.40 (0.04)	16.66 (0.10)	16.43 (0.09)	17.13 (0.18)	16.24 (0.05)
	BO	83.44 (0.07)	83.00 (0.16)	83.21 (0.13)	81.93 (0.34)	83.68 (0.12)
	TBO	0.17 (0.04)	0.33 (0.07)	0.32 (0.05)	0.94 (0.17)	0.08 (0.07)
SANK	NBO	16.42 (0.07)	16.44 (0.05)	16.35 (0.17)	17.62 (0.12)	16.29 (0.08)
	BO	83.40 (0.13)	83.39 (0.10)	83.30 (0.20)	80.61 (0.38)	83.63 (0.14)
	TBO	0.19 (0.06)	0.17 (0.06)	0.30 (0.09)	1.67 (0.23)	0.09 (0.06)
SAK	NBO	16.62 (0.10)	16.60 (0.09)	16.48 (0.08)	19.18 (0.35)	16.26 (0.06)
	BO	83.02 (0.19)	83.13 (0.16)	83.28 (0.16)	76.28 (0.76)	83.67 (0.08)
	TBO	0.36 (0.09)	0.27 (0.07)	0.25 (0.08)	4.02 (0.41)	0.08 (0.03)

Standard deviations are reported in parenthesis.

**Figure 7:**
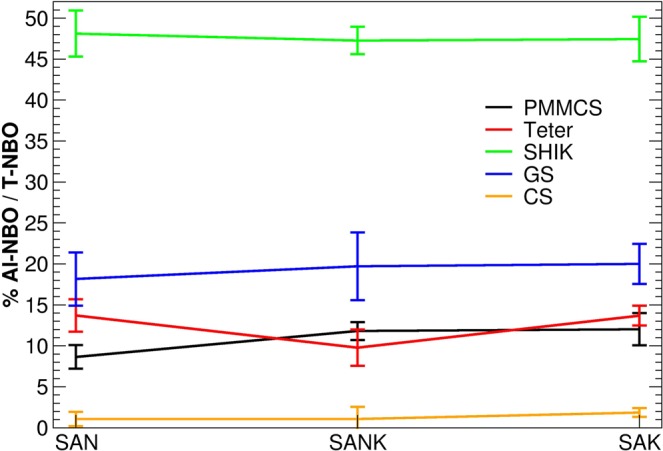
Percentage of Al-NBO bonds on the total amount of T-NBO bonds (where T = Al and Si) as a function of the glass composition and interatomic potential used.

In aluminosilicate glasses, NBOs are usually bounded to silicon rather than aluminum since the latter is embedded in a tetrahedral environment (in aluminosilicate glasses with Na/Al < 1.6)^48^, which already bears a negative charge and is compensated by modifier cations. This has been also confirmed by NMR experiments coupled with DFT calculations^28,33^. We thus monitor the amount of NBO connected to aluminum obtained by the interatomic potentials as depicted in Fig. 7, where the percentage of Al-NBO on the total amount of T-NBO bonds (T = Al and Si) is reported as a function of the glass composition. Interestingly, very different results are observed among the potentials. In the case of the SHIK potential, about 50% of NBO are connected to Al (with distances of 1.74 Å), whereas less than 2% NBO are connected to Al when the CS potential is employed. The other potentials are in-between the two potentials: the GS models provide about 20% of NBO connected to Al, whereas the Teter and PMMCS models possess 10–15% of NBO connected to Al. No particular trends are observed with glass composition, denoting that the substitution of Na with K does not alter the amount of NBO and their distributions. Later on, it will be shown that the lowest NBO-Al connection in the CS model relates to the reproducibility for MAE.

#### Intermediate range order

One of the most used parameters to characterize the medium range order in glasses is the so-called Q^n^ species distribution (where Q stands for quaternary species and *n* is the number of BO connected to it), which is assumed to represent the network polymerization. Table 6 reports the Q^n^ distributions and network connectivity (NC, defined as the average number of BOs per network-forming) of silicon and aluminum in the three glasses obtained using the different interatomic potentials. The silicon network is dominated by Q^4^ and Q^3^ species. All the potentials provide similar results (around 56–61% of Q^4^ for SAN) except for the SHIK one, which possesses a higher amount of Q^4^ species (67%). The more polymerized silicate networks obtained in the SHIK models are consistent with the fact that 50% of NBOs are connected to aluminum in the models. No significant difference in the Q^n^ species distribution is observed among the three glass compositions for the Teter and CS potentials. On the contrary, the GS and, to a lesser extent, PMMCS potentials yield a reduction of the Q^3^ species with increasing of potassium.

**Table 6:** Q^n^ species distribution and network connectivity (NC) for silicon and aluminium in the SAN, SANK and SAK glasses simulated with the different interatomic potentials (PMMCS, Teter, SHIK,GS and CS).

%			PMMCS	Teter	SHIK	GS	CS
SAN	Q^n^ Si	1	0.05 (0.10)	0.24 (0.12)	0.17 (0.09)	0.65 (0.16)	0.04 (0.08)
		2	3.95 (0.52)	4.80 (0.47)	3.30 (0.16)	5.64 (0.19)	2.20 (0.58)
		3	37.33 (1.08)	34.91 (1.55)	29.29 (0.43)	32.42 (1.42)	41.56 (0.82)
		4	58.65 (0.57)	60.05 (1.00)	67.22 (0.61)	61.27 (1.27)	56.04 (0.27)
		*NC*	*3.545*	*3.548*	*3.635*	*3.543*	*3.533*
	Q^n^ Al	1			0.24 (0.16)		
		2	0.02 (0.01)	0.19 (0.17)	2.84 (0.98)	0.25 (0.32)	
		3	4.61 (0.83)	7.81 (1.53)	27.44 (2.66)	9.84 (1.45)	0.53 (0.41)
		4	95.09 (0.89)	91.92 (1.35)	69.35 (2.63)	89.67 (1.65)	99.48 (0.41)
		*NC*	*3.942*	*3.915*	*3.656*	*3.887*	*3.995*
SANK	Q^n^ Si	1	0.12 (0.09)	0.29 (0.13)	0.10 (0.01)	1.63 (0.37)	0.01 (0.01)
		2	5.03 (0.39)	4.55 (0.23)	3.61 (0.32)	7.68 (1.06)	2.31 (0.45)
		3	34.47 (1.05)	35.31 (0.65)	28.95 (0.88)	25.49 (1.00)	41.55 (1.03)
		4	60.36 (0.69)	59.84 (0.50)	67.31 (0.62)	64.95 (0.69)	56.07 (0.57)
		*NC*	*3.550*	*3.547*	*3.634*	*3.532*	*3.536*
	Q^n^ Al	1			0.08 (0.16)		
		2	0.01 (0.00)	0.02 (0.03)	2.22 (0.58)	0.72 (0.64)	
		3	6.29 (0.66)	5.42 (1.03)	28.09 (1.84)	10.66 (1.97)	0.56 (0.73)
		4	93.48 (0.77)	94.43 (1.07)	69.37 (1.93)	87.59 (2.87)	99.44 (0.73)
		*NC*	*3.928*	*3.940*	*3.662*	*3.838*	*3.994*
SAK	Q^n^ Si	1	0.18 (0.12)	0.24 (0.10)	0.07 (0.09)	2.60 (0.21)	0.00 (0.00)
		2	5.47 (0.50)	5.08 (0.58)	2.73 (0.66)	9.07 (1.00)	2.54 (0.40)
		3	33.96 (0.94)	34.16 (1.01)	31.01 (0.76)	21.97 (0.73)	40.90 (0.79)
		4	60.40 (0.53)	60.51 (0.43)	66.19 (0.33)	65.59 (1.08)	56.45 (0.39)
		*NC*	*3.546*	*3.549*	*3.633*	*3.490*	*3.536*
	Q^n^ Al	1			0.16 (0.18)		
		2	0.07 (0.10)	0.17 (0.17)	2.53 (1.12)	1.52 (0.31)	
		3	6.50 (1.19)	7.82 (1.05)	28.19 (1.09)	10.77 (1.27)	0.91 (0.27)
		4	93.33 (1.19)	92.00 (0.89)	69.25 (2.01)	86.07 (1.04)	99.09 (0.27)
		*NC*	*3.930*	*3.918*	*3.666*	*3.798*	*3.991*

Standard deviations are reported in parenthesis.

As expected from the previous analysis on the percentage of the Al-NBO bonds with respect to the total amount of T-NBO as reported in Fig. 7, the SHIK potential yields the highest amount of Q^3^ species bound to Al (~28%) while the CS does the lowest amount (less than 1%). As a result, the order of the network connectivity is CS (3.99) > PMMCS ≈ Teter (3.92–3.94) > GS (~3.80) > SHIK (3.66). The SHIK potential provides equal NC for silicon and aluminum. This is probably because they bear similar charges (q_Si_ = 1.7755, q_Al_ = 1.6334) and this seems to be a disadvantage of this potential.

The simulation result on the Al-NBO by the CS potential agrees well with experimental observation. In fact, NMR experiments suggest that a sufficient concentration of modifiers to balance negatively charged AlO_4_^−^ units in alkali aluminosilicate glasses with [Al]/[Si] < 1 and aluminum is thus tetrahedrally coordinated and more reluctant to accept NBOs compared to the other network formers, such as silicon (see ref. ^49^ and references therein). Therefore, when the [Na_2_O]/[Al_2_O_3_] ratio is higher than one, as in this study, the NC of silicon is always lower than that of aluminum^28^.

**Figure 8:**
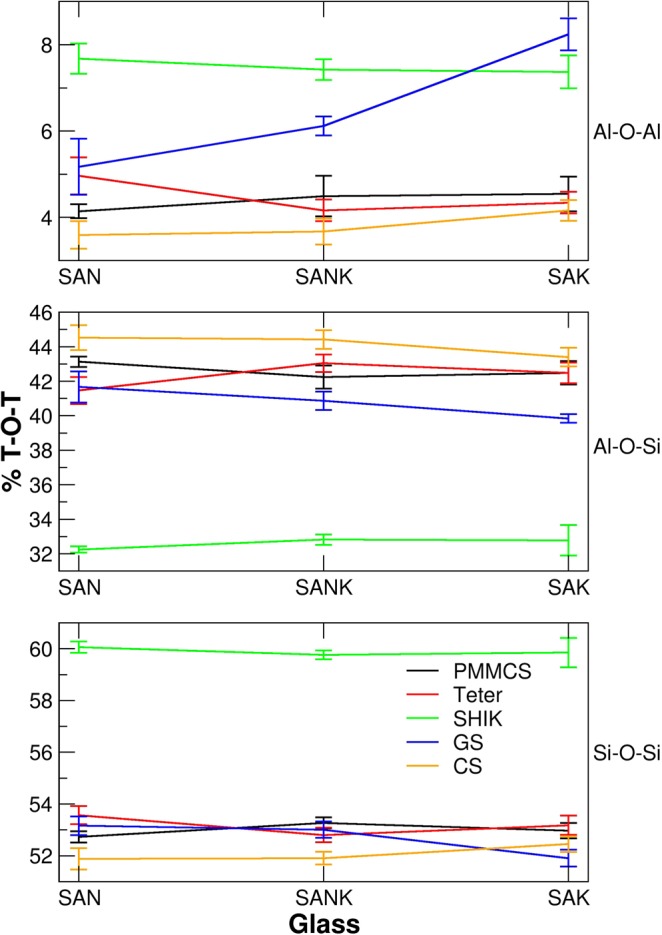
Percentage of T-O-T bridges found in the glasses with the different potentials. The percentage is computed with respect to the total amount of oxygens (BO and NBO) in the glasses.

Moreover, to avoid negative charge-accumulation in the structure, Si-O-Al^[4]^ bond should be preferred rather than Al^[4]^-O-Al^[4]^, and the latter is absent except for the case of aluminosilicate glasses containing high field strengths cations such as Rare-Earth elements^50^. To investigate the degree of intermixing between network formers, Si and Al, the percentages of T-O-T bridges were analyzed for the three glasses obtained by the different potentials. According to the plots in Fig. 8, several interesting observations are worth to note: 1) the SHIK potential provides structure with less intermixing between network formers presenting the highest amount of Si-O-Si (~60%) and Al-O-Al (~7.5%) bonds and lower amount of Si-O-Al (~32%) bonds; 2) clear trends on the T-O-T bonds among the glasses is not observed for the most of the interatomic potentials except for the GS one for which an increment of Al-O-Al and a decrement of the Si-O-Al and Si-O-Si bonds are observed when sodium is gradually replaced by potassium; 3) PMMCS and Teter potentials provide similar amounts of T-O-T bonds but different trends with the Na/K substitution. 4) More importantly, good agreement with NMR experiments on aluminosilicate glasses^50^ can be found for the CS potential, which provides the smallest amount of Al-O-Al (3–4%) and higher amount of Al-O-Si bonds (~44%) and no clustering of the same sort of alkaline ions. That means that the CS potential produces glasses with greater intermixing between network formers and alkaline cations in the percolation channels. We expect that the accurate structure generated by the CS potential would be a key to the reproducibility of the MAE by the potential.

**Figure 9:**
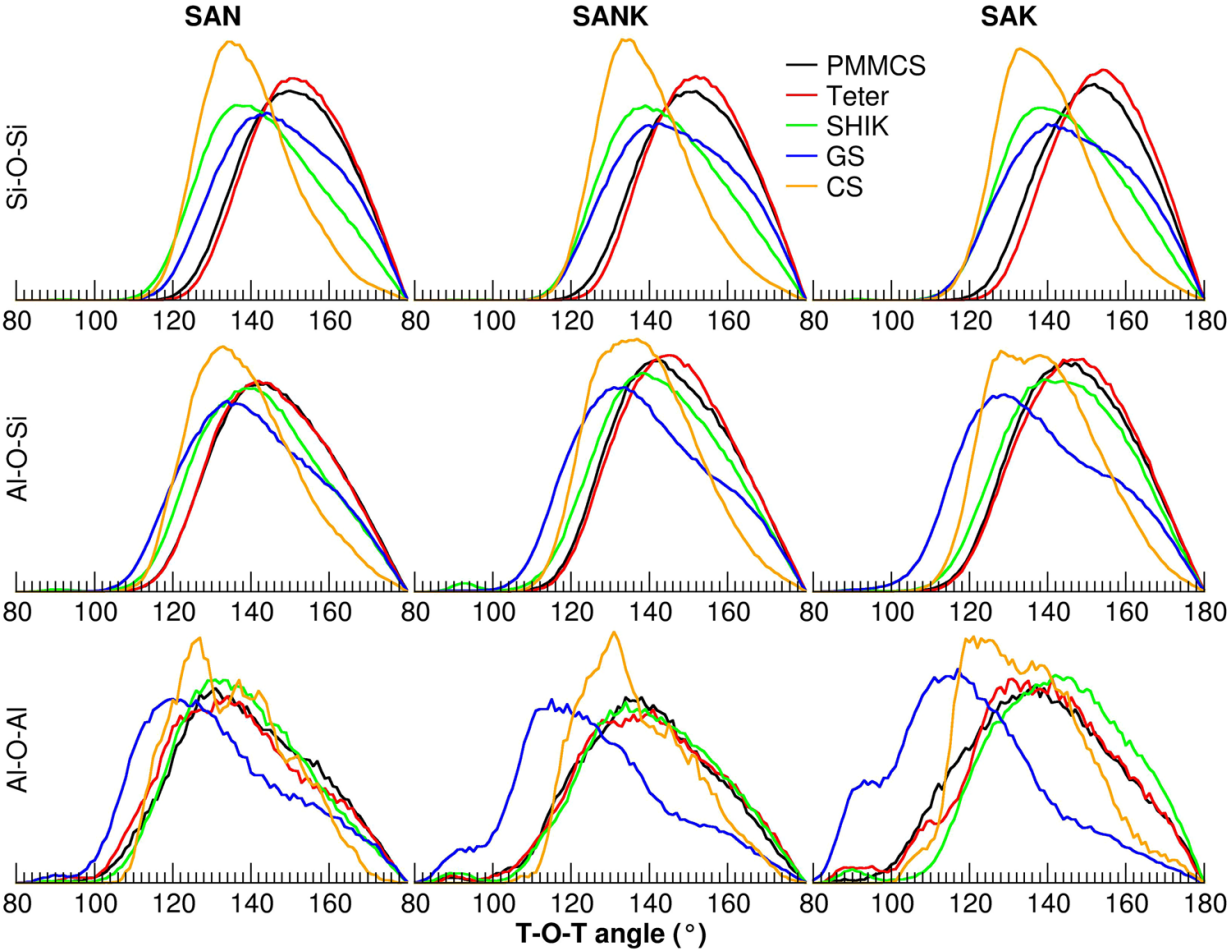
Bond angle distributions for different T-O-T angles of the three glasses studied with different interatomic potentials.

The different interatomic potentials provide not only different amount of T-O-T bonds but also quite different T-O-T bond angle distributions (BAD), as shown in Fig. 9. In particular, the CS potential gives the narrower distributions and smaller angles for the Si-O-Si and Al-O-Si bonds thanks to the inclusion of polarizability of oxygen ions. In general, the order of the peak position is the CS < SHIK < GS < PMMCS ≈ Teter for the Si-O-Si angles and CS < GS < SHIK < PMMCS ≈ Teter for the Al-O-Si angle. In general, the Si−O−Si angles slightly decrease when sodium is substituted by potassium in agreement with the observation of previous ^17^O NMR experiments^51^ and ab initio MD simulations^52^ that showed that the Si-O-Si angles decrease with increasing ionic radius of the alkaline cations.

In previous works^28,53^, we have demonstrated that the width of the BAD of T-O-T angles affects the shape of the NMR spectra. It is thus we can infer that the glass models, which provide narrower BAD distributions, constructed by the CS potential would improve the agreement between experiments and MD simulations for the NMR spectra. Note here that the peak of the Al-O-Si BAD splits into two peaks with the substitution of Na with K when the CS model is applied. This trend is not observed with the other potentials.

The T-O-T BADs would reflect on the cation-cation pair distribution functions, which are shown only for the SANK glass in Fig. 5, and those of SAN and SAK glasses are drawn in Figures S8 and S9 of the ESI. Some interesting features worth noting are: (1) the Al-Na PDF obtained using the SHIK potential presents a double first peak, representing two possible sites for Na around AlO_4_ tetrahedra; the first peak is associated with Na acting as a modifier connected to Al-NBO, whereas the second peak is associated with Na acting as a compensator connected to Al-BO-T bonds. The double peak is not observed for the Al-K PDFs, suggesting that a sodium ion acts as both of a modifier and a charge compensator for aluminum, whereas a potassium ion prefers to act as a charge compensator only in the SANK glass. (2) The alkali-alkali PDFs computed with the PMMCS, Teter and CS potentials present well-defined peaks extending over three alkali coordination shells, implying the formation of percolation channels with mixed alkali ions. This observation is in agreement with spin echo and REDOR experiments^18^. The alkaline ions ordering is also observed in the models by the SHIK potential for Na-Na and Na-K PDFs but not for the K-K PDF. The K-K PDF has a broader first peak locating at larger distance (4.2 Å) than that observed with the CS potential (3.8 Å), and the first peak intensity is slightly less than that of the second peak. These results suggest that the SHIK potential distributes potassium ions more homogeneously than the CS one in the percolation channels. (3) The GS potential provides longer Al-K and Si-K distances and strong first K-K peak at shorter distance compared with the other potentials, denoting a more propensity of K clustering in the structure.

**Figure 10:**
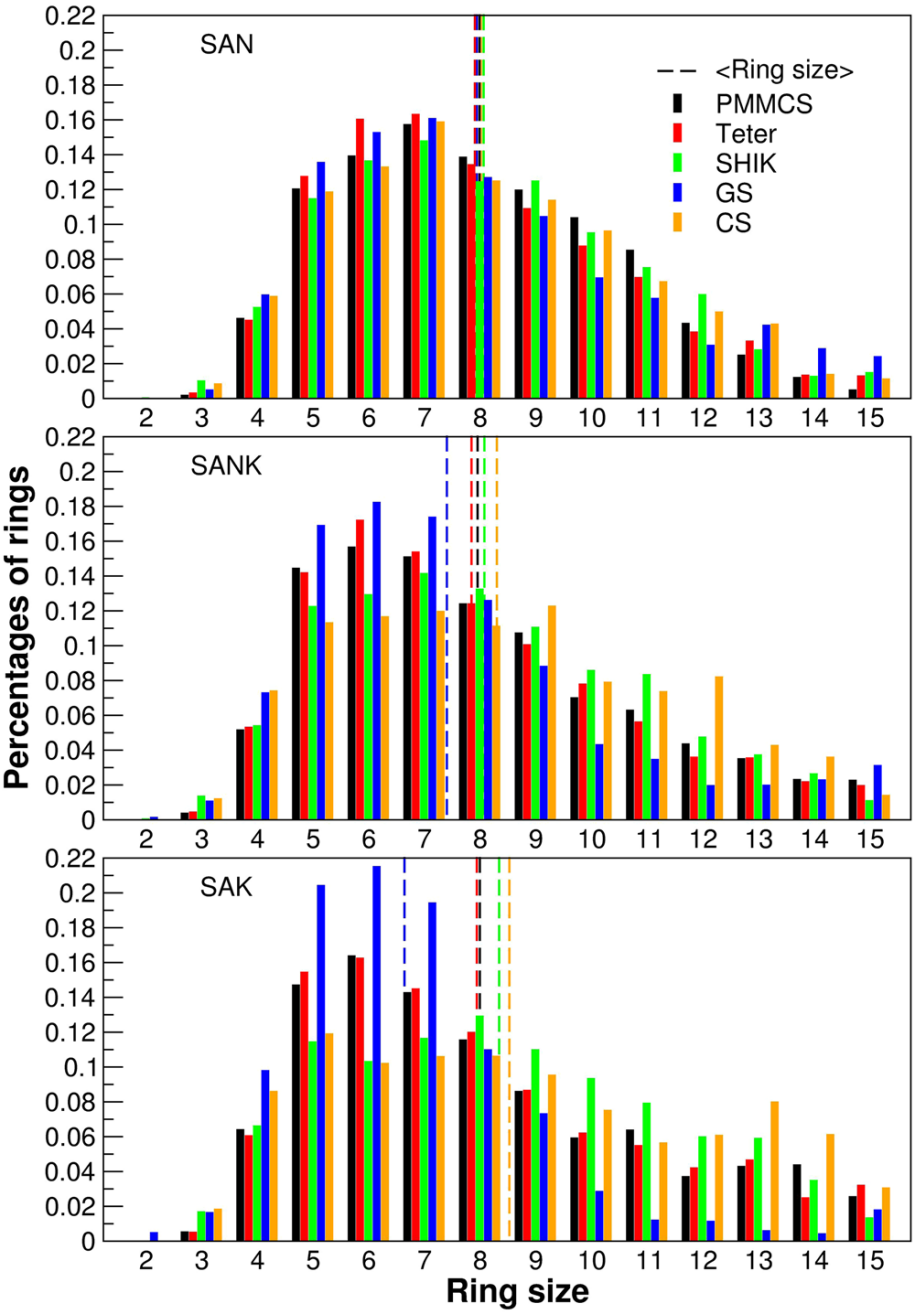
Ring size distributions of glasses obtained by using different interatomic potentials and relative average ring size represented by dashed lines.

Here we again discuss the ring size distributions obtained by the five potentials, according to Fig. 10 and Table 4. A shift of the average ring size towards smaller rings is observed by increasing the K concentration in the structure for the PMMCS, Teter and GS potentials, whereas the SHIK and CS models show a shift in the opposite direction. As discussed above, the larger rings contribute to form the percolation channels of the alkaline ions suitable for their diffusion. It is worth noting that the SHIK and CS potentials are the ones that give the narrower inter-tetrahedra Si-O-Si angles as drawn in Fig. 8. Indeed, this angle significantly influences the ring size distributions and the medium range structure since the smaller inter-tetrahedra angle implies that a ring accommodating large alkaline ions like potassium should be composed of more tetrahedral units. Another way of saying, alkali cations would be accommodated by smaller rings in the glass structures constructed by the PMMCS, Teter and GS potentials because they possess larger Si-O-Si inter-tetrahedra angle. In summary, the microstructure with the narrowest Si-O-Si angle results the largest ring size, distinguishes the CS potential from the other force field models and allows to simulate MAE appropriately.

To conclude the discussions, the snapshots of the SANK glass generated using the CS, SHIK and GS potentials are compared in Fig. 4. In the top panel of the figure, silicon and aluminum atoms are represented as yellow and magenta tetrahedra. Aluminum clustering and silica richer zones are evident for the SHIK and GS models, whereas in the CS model a more uniform distribution of the two cations can be observed. The lower panel of Fig. 4 shows the distribution of sodium (cyan spheres) and potassium (green spheres) ions. The visual analysis of alkali distributions can be quantified by the cation-cation coordination numbers listed in Table 7. In the CS model, sodium slightly prefers to locate in the vicinity of silicon rather than aluminum (CN(Al-Na)/CN(Si-Na) = 0.92), whereas potassium surrounds both network formers equally (CN(Al-K)/CN(Si-K) = 1.03) and both cations form the percolation channels. In the SHIK model, both modifiers prefer to surround aluminum rather than silicon (CN(Al-Na)/CN(Si-Na) = 1.18 and CN(Al-K)/CN(Si-K) = 1.17), which indeed present the higher network connectivity. As observed above, in the GS model, the alkaline cations (especially potassium ions) segregates in large regions separated from both silicon and aluminum ions. The models generated with PMMCS and Teter potentials are relatively similar with that produced by the CS potential and thus not drawn in Fig. 4.

**Table 7:** Cation-cation average coordination numbers of the first coordination sphere in the SAN, SANK and SAK glasses simulated with the different interatomic potentials (PMMCS, Teter, SHIK, GS and CS). Standard deviations are reported in parenthesis.

	<CN>	PMMCS	Teter	SHIK	GS	CS
SAN	Al-Na	4.88 (0.05)	4.87 (0.06)	4.54 (0.04)	4.52 (0.05)	4.52 (0.11)
	Si-Na	4.77 (0.02)	4.60 (0.04)	4.00 (0.03)	4.45 (0.02)	4.59 (0.02)
	Na-Na	4.56 (0.02)	5.50 (0.05)	4.82 (0.03)	6.05 (0.03)	5.10 (0.04)
SANK	Al-Na	2.34 (0.06)	2.17 (0.05)	2.07 (0.11)	2.34 (0.05)	1.81 (0.06)
	Al-K	2.51 (0.06)	2.77 (0.04)	3.00 (0.04)	2.17 (0.07)	2.65 (0.09)
	Si-Na	2.22 (0.03)	2.16 (0.01)	1.75 (0.02)	2.22 (0.04)	1.97 (0.03)
	Si-K	2.45 (0.03)	2.59 (0.03)	2.57 (0.01)	2.37 (0.03)	2.55 (0.02)
	Na-Na	2.06 (0.04)	2.18 (0.08)	2.55 (0.12)	1.42 (0.07)	2.17 (0.06)
	Na-K	2.30 (0.03)	2.38 (0.06)	2.40 (0.03)	2.10 (0.07)	2.35 (0.02)
	K-K	2.75 (0.07)	2.49 (0.08)	2.24 (0.09)	4.02 (0.05)	2.70 (0.04)
SAK	Al-K	5.25 (0.02)	5.31 (0.09)	5.38 (0.05)	3.41 (0.27)	4.63 (0.05)
	Si-K	4.79 (0.01)	4.89 (0.05)	4.76 (0.02)	4.52 (0.11)	4.76 (0.06)
	K-K	5.16 (0.03)	4.48 (0.06)	5.21 (0.03)	5.84 (0.11)	5.36 (0.04)

## Conclusions

In order to reproduce the non-linear variations of the glass properties, such as glass transition temperature and ionic conductivity, by mixing of alkaline ions, Na and K, using molecular dynamics simulations, we benchmarked a variety of major force field models available in the literature to simulate aluminosilicate glasses. Four types of interatomic potentials (PMMCS, Teter, GS and SHIK) based on the rigid ionic model in addition to the polarizable core-shell model (CS) have been examined. As a result, we found that the CS model is the only one that was able to reproduce the alkaline mixing effect in both ionic conductivity and glass transition temperature. Regarding the ionic conductivity, the CS potential is able to reproduce not only the mixed alkali effect but also the increased ionic conductivity of single SAK glass compared to the SAN glass at higher temperature.

According to the extensive investigations on the microstructure, the mixed alkali effect is successfully explained by the formation of percolation channels containing both Na and K ions in the glass model constructed with the CS potential. Further, it was unraveled that the narrowest inter-tetrahedra Si-O-Si angle observed in the CS models contributes to form larger rings, which accommodate alkaline ions, resulting in the alkaline percolation channels as the origin of the mixing alkaline effect. Indeed, the alkaline ion jump analysis using the van Hove correlation function clearly demonstrates that the alkaline ions locate in different local environments and obstruct each other in the channel, restricting their mobility in the SANK glass. The other intriguing behavior observed in ion conductivity is that the SAK glass possesses the higher ionic conductivity in comparison with the SAN glass only at high temperature. The CS potential solely succeeds to demonstrate the phenomenon thanks to the easier expansion of percolation channels, which are created by the larger and more flexible rings, in the SAK glass.

The PMMCS and Teter potentials provide structures similar to the ones obtained with the CS potential, but they develop different bond angles and rings size distributions, and they are thus not able to reproduce the right trends of the MAE on the glass transition temperature and the higher mobility of potassium at high temperature. The SHIK and GS potentials provide significant differences in the glass structures with respect to those generated with the CS model. In particular, we observed a propensity to form clusters of the same alkaline cations (especially potassium) and a lower degree of intermixing between network formers cations (Si and Al), which can be the possible reasons of their failure to reproduce the ionic conductivity trends.

Indeed, the structures generated with the SHIK potential have higher amount of Al-O-Al bonds, and NBOs are equally partitioned by Al and Si. Because of the excess of negative charge held by AlO_4_ units, the alkali cations (especially K ions) are strongly attracted by such units and their diffusion highly hindered, misleading the resistivity change with the potassium ion increasing. Exactly the opposite trend has been found for the GS potential that provides extremely heterogeneous structures with the larger clustering of potassium ions and segregations of the SiO_4_ and the AlO_4_ rich zones.

## Supplementary information

Electronic Supplementary Material.

## Data Availability

The data that supports the findings of this work are available from the corresponding author upon reasonable request.
