# Supplementary material for: Structural origins of the Mixed Alkali Effect in Alkali Aluminosilicate Glasses: Molecular Dynamics Study and its Assessment
Source: Sci Rep. 2020 Feb 19;10:2906. doi: 10.1038/s41598-020-59875-7 (PMC7031271; doi:10.1038/s41598-020-59875-7)
Supplement: Supplementary file 1 — Electronic Supplementary Material. [file 41598_2020_59875_MOESM1_ESM.pdf]

Supporting Information

Structural origins of the Mixed Alkali Effect in Alkali Aluminosilicate Glasses: Molecular Dynamics Study and its Assessment

Federica Lodesani,[a] Maria Cristina Menziani,[a] Hiroyuki Hijiya,[b] Yoichi Takato,[c] Shingo Urata[c] and Alfonso Pedone [a,*]

[a)]Department of Chemical and Geological Sciences, University of Modena and Reggio Emilia, via G. Campi 103, 41125, Modena, Italia

[b)] Materials Integration Laboratories, AGC Inc., Yokohama, Kanagawa 221-8755, Japan

[c)]Innovative Technology Laboratories, AGC Inc., Yokohama, Kanagawa 221-8755, Japan

Force Fields Parameters.

Table S1: Rigid Ionic model: Morse and Buckingham interatomic potential parameters used for molecular dynamics simulations.

Morse parameters				
**Atom Pairs**	**D_{ij} [eV]**	**a_{ij} [Å^{-2}]**	**r_0[Å]**	**B_{ij} [eV Å^{12}]**
$O^{-1.2}$-$O^{-1.2}$	0.042395	1.379316	3.618701	22.0
$Si^{+2.4}$-$O^{-1.2}$	0.340554	2.006700	2.100000	1.0
$Na^{+0.6}$-$O^{-1.2}$	0.023363	1.763867	3.006315	5.0
$K^{+0.6}$-$O^{-1.2}$	0.011612	2.062605	3.305308	5.0
Buckingham parameters				
**Atom Pairs**	**A_{ij} [eV]**	**ρ_{ij} [Å^{-2}]**	**C_{ij} [eV Å^6]**	**B_{ij} [eV Å^{12}]**
$Al^{+1.8}$-$O^{-1.2}$	12201.41	0.1956	31.997	15.0

Table S2. GS Interatomic potential parameters. (q_O=-0.945, q_{Si}=1.89, q_{Al}=1.4175, q_{Na}=0.4725, q_K=0.4725)

**Atom Pairs**	**A_{ij} (eV)**	**ρ_{ij} (Å)**	**C_{ij} (eV Å^6)**
O-O	9022.79	0.265	85.0921
Si-O	50306.10	0.161	46.2978
Al-O	28538.42	0.172	34.5778
Na-O	120303.80	0.17	0.00
K-O	2284.77	0.29	0.00

Table S3. Du Interatomic potential parameters. (q_O=-1.2, q_{Si}=2.4, q_{Al}=1.8, q_{Na}=0.6, q_K=0.6)

**Pairs**	**A_{ij} (eV)**	**ρ_{ij} (Å)**	**C_{ij} (eV Å^6)**	**B_{ij} (eV Å^{nij})**	**D_{ij} (eV Å^2)**	**n_{ij}**	**r_0**
O-O	2029.2204	0.343645	192.58	45.868	-0.333	3.402	1.903
Si-O	13702.9050	0.193817	54.681	28.950	-3.059	3.932	1.168
Al-O	12201.4170	0.195628	31.997	51.728	-10.197	3.179	1.024
Na-O	4383.7555	0.243838	30.700	48.264	-4.730	2.893	1.172
K-O	20526.9720	0.233708	51.489	311.662	-78.964	2.459	0.949

Table S4. SHIK interatomic potential parameters (q_{Si}=1.7755, q_{Al}=1.6334, q_{Na}=0.6018, q_K=0.6294)

Atom Pairs	A_{ij} (eV)	ρ_{ij} (Å)	C_{ij} (eV Å^6)	D_{ij} (eV Å^2)
O-O	1120.5	2.8927	26.132	16800
O-Si	23108	5.0979	139.70	66
Si-Si	2798.0	4.4073	0.0	3423204
O-Na	1127566	6.8986	40.562	16800
Si-Na	495653	5.4151	0.0	16800
Na-Na	1476.9	3.4075	0.0	16800
O-K	219750	5.2494	111.97	16800
Si-K	550659	4.8283	0.0	16800
K-K	1177.8	2.7363	0.0	16800
O-Li	6745.2	4.9120	41.221	70
Si-Li	17284	4.3848	0.0	16800
Li-Li	2323.8	3.9129	0.0	3240
O-Al	21740	5.3054	65.815	66
Al-Al	1799.1	3.6778	100.0	16800

Table S5. Shell model interatomic potential: analytic functions and parameters.

		Buckingham	
**Pairs**	**A (eV)**	**ρ (Å)**	**C (eV Å^6)**
O_s-O_s	22764.30	0.1490	27.88
Si-O_s	1283.91	0.32052	10.661580
K-O_s	7338.603	0.263891	0.000000
Na-O_s	56465.345	0.193931	0.000000
Al-O_s	1460.3000	0.29912	0.0000000
		Three-body potential	
	**k_b (eV rad^{-2})**	**θ_0 (deg)**	**ρ (Å)**
O-Si-O	100.0	109.47	1.0
O-Al-O	100.0	109.47	1.0
		Core-shell potential	
	**k_s (eV Å^{-2})**	**Y(e)**	
O_c-O_s	74.92	-2.8482	

Table S6. Lattice parameters and bond distances for the $K_2Si_2O_5$, $K_6Si_2O_7$ and $KAlSiO_4$ crystals computed using the core-shell interatomic potentials reported in Table S5.

	**$K_2Si_2O_5$**			**$K_6Si_2O_7$**			**$KAlSiO_4$**		
	**exp**	**calc**	**%err**	**exp**	**calc**	**%err**	**exp**	**calc**	**%err**
a **(Å)**	16.3224	16.8043	2.95	6.4580	6.3539	-1.61	5.1610	5.1715	0.20
b **(Å)**	11.2430	11.2839	0.36	8.8870	8.8309	-0.63	5.1610	5.1715	0.20
**c (Å)**	9.9190	10.0205	1.02	10.879	10.834	-0.42	8.6930	8.6747	-0.21
**Vcell**	1636.459	1688.671	3.19	511.45	500.21	-2.20	200.52	200.91	0.19
**<K-O>**	2.841	2.811	-1.1	2.781	2.757	-0.9	2.968	2.980	0.4
**<Si-O>**	1.624	1.618	-0.4	1.635	1.627	-0.5	1.608	1.610	0.12
**<Al-O>**	-	-	-	-	-	-	1.735	1.725	-0.6

Table S7. Calculated mobility, μ, of Na and K ions along X direction, with the five potentials for the three glasses and three different temperatures and relative fraction of mobile ions

	μ ·10^{-6} (cm^2 / V · s) X direction							
	**T (K)**	**Cation**	**SANK0**	**f**	**SANK12.5**	**f**	**SANK25**	**f**
**PMMCS**	**550**	**Na**	0.92 (0.04)	0.426	0.91 (0.21)	0.426		
		**K**			0.77 (0.06)	0.495	3.00 (0.54)	0.652
	**650**	**Na**	2.16 (0.23)	0.665	2.30 (0.41)	0.667		
		**K**			1.42 (0.43)	0.670	6.62 (0.93)	0.821
	**800**	**Na**	7.83 (0.38)	0.927	9.22 (0.37)	0.924		
		**K**			5.76 (1.88)	0.903	18.43 (2.90)	0.943
	**950**	**Na**	24.25 (3.48)	0.992	25.50 (1.09)	0.989		
		**K**			15.72 (4.22)	0.984	34.78 (2.72)	0.981
**Teter**	**550**	**Na**	1.04 (0.12)	0.465	0.62 (0.17)	0.342		
		**K**			0.34 (0.03)	0.247	1.09 (0.02)	0.426
	**650**	**Na**	2.81 (0.93)	0.732	1.63 (0.14)	0.607		
		**K**			0.64 (0.15)	0.447	2.43 (0.16)	0.619
	**800**	**Na**	8.91 (2.48)	0.928	4.95 (0.46)	0.871		
		**K**			2.08 (0.80)	0.748	7.59 (1.42)	0.837
	**950**	**Na**	25.74 (5.64)	0.990	14.57 (1.58)	0.969		
		**K**			5.83 (1.01)	0.887	13.21 (1.03)	0.930
**SHIK**	**650**	**Na**	1.11 (0.32)	0.568	0.29 (0.04)	0.322		
		**K**			0.09 (0.03)	0.131	0.16 (0.03)	0.141
	**800**	**Na**	5.04 (0.97)	0.887	1.68 (0.17)	0.713		
		**K**			0.35 (0.02)	0.362	0.56 (0.10)	0.392
	**950**	**Na**	14.48 (0.76)	0.981	5.28 (0.55)	0.933		
		**K**			1.08 (0.26)	0.651	1.53 (0.57)	0.638
**GS**	**370**	**Na**	2.46 (0.55)	0.505	0.59 (0.16)	0.288		
		**K**			6.10 (1.41)	0.738	45.15 (5.23)	0.880
	**420**	**Na**	3.77 (0.45)	0.650	1.83 (0.35)	0.483		
		**K**			11.19 (1.31)	0.852	70.97 (3.27)	0.929
	**500**	**Na**	9.07 (1.66)	0.809	6.23 (0.55)	0.695		
		**K**			24.36 (4.53)	0.933	121.94 (28.80)	0.958
**CS**	**950**	**Na**	1.11 (0.23)	0.595	0.33 (0.06)	0.372		
		**K**			0.28 (0.05)	0.363	0.77 (0.03)	0.515
	**1190**	**Na**	8.52 (1.77)	0.956	4.00 (0.25)	0.899		
		**K**			4.10 (0.38)	0.899	13.90 (1.09)	0.943
	**1350**	**Na**	15.35 (2.59)	0.991	8.87 (0.31)	0.972		
		**K**			9.36 (1.36)	0.955	23.37 (1.56)	0.976

Table S1. Bond distances and coordination numbers for the SAN, SANK and SAK glasses simulated with the different interatomic potentials (PMMCS, Teter, SHIK, GS and CS).

			**PMMCS**		**Teter**		**SHIK**		**GS**		**CS**	
			**Distance (Å)**	<CN>	**Distance (Å)**	<CN>	**Distance (Å)**	<CN>	**Distance (Å)**	<CN>	**Distance (Å)**	<CN>
**SAN**	**Al**	**O**	1.78	4.00	1.78	3.99	1.76	4.00	1.74	4.00	1.73	4.00
		**BO**	1.78	3.92	1.78	3.84	1.77	3.62	1.74	3.72	1.73	3.98
		**NBO**	1.73	0.04	1.73	0.07	1.74	0.34	1.72	0.10	1.70	0.01
	**Si**	**O**	1.61	4.00	1.60	4.00	1.62	4.00	1.63	4.00	1.62	4.00
		**BO**	1.62	3.54	1.60	3.54	1.62	3.62	1.63	3.52	1.62	3.54
		**NBO**	1.55	0.45	1.55	0.45	1.60	0.36	1.62	0.46	1.55	0.46
	**Na**	**O**	2.40	5.57	2.42	5.55	2.30	5.42	2.47	5.37	2.37	5.52
		**BO**	2.48	3.88	2.52	3.56	2.35	3.64	2.55	3.24	2.43	3.70
		**NBO**	2.28	1.69	2.34	1.98	2.28	1.77	2.43	2.11	2.29	1.82
**SANK**	**Al**	**O**	1.78	4.00	1.78	4.00	1.76	4.00	1.74	4.01	1.73	4.00
		**BO**	1.78	3.90	1.78	3.91	1.76	3.64	1.74	3.57	1.73	3.97
		**NBO**	1.73	0.06	1.72	0.05	1.74	0.33	1.72	0.12	1.70	0.01
	**Si**	**O**	1.60	4.00	1.60	4.00	1.62	4.00	1.63	4.00	1.62	4.00
		**BO**	1.62	3.55	1.60	3.54	1.62	3.62	1.63	3.49	1.62	3.54
		**NBO**	1.55	0.45	1.55	0.45	1.60	0.36	1.62	0.47	1.55	0.46
	**Na**	**O**	2.36	5.36	2.41	5.36	2.29	5.19	2.46	5.62	2.34	5.46
		**BO**	2.48	3.57	2.52	3.20	2.34	3.13	2.52	3.61	2.43	3.44
		**NBO**	2.26	1.79	2.34	2.16	2.27	2.04	2.42	1.96	2.28	2.02
	**K**	**O**	2.74	9.24	2.81	9.82	2.72	9.93	3.02	7.71	2.72	9.51
		**BO**	2.82	6.67	2.87	7.39	2.76	7.77	3.19	3.23	2.82	7.37
		**NBO**	2.67	2.56	2.74	2.41	2.67	2.13	2.96	3.30	2.60	2.14
**SAK**	**Al**	**O**	1.78	4.00	1.78	3.99	1.76	4.00	1.74	4.00	1.73	4.00
		**BO**	1.78	3.86	1.78	3.86	1.77	3.64	1.74	3.14	1.73	3.97
		**NBO**	1.73	0.06	1.72	0.07	1.74	0.33	1.72	0.13	1.69	0.01
	**Si**	**O**	1.62	4.00	1.60	4.00	1.62	4.00	1.63	4.00	1.62	4.00
		**BO**	1.62	3.54	1.60	3.54	1.62	3.62	1.63	3.38	1.62	3.54
		**NBO**	1.55	0.45	1.55	0.45	1.59	0.37	1.62	0.51	1.54	0.46
	**K**	**O**	2.72	8.79	2.78	9.05	2.71	9.36	2.98	7.39	2.69	8.97
		**BO**	2.83	6.27	2.88	6.49	2.75	7.19	3.10	3.63	2.80	6.72
		**NBO**	2.65	2.50	2.72	2.54	2.65	2.16	2.93	3.56	2.58	2.25

Figures.

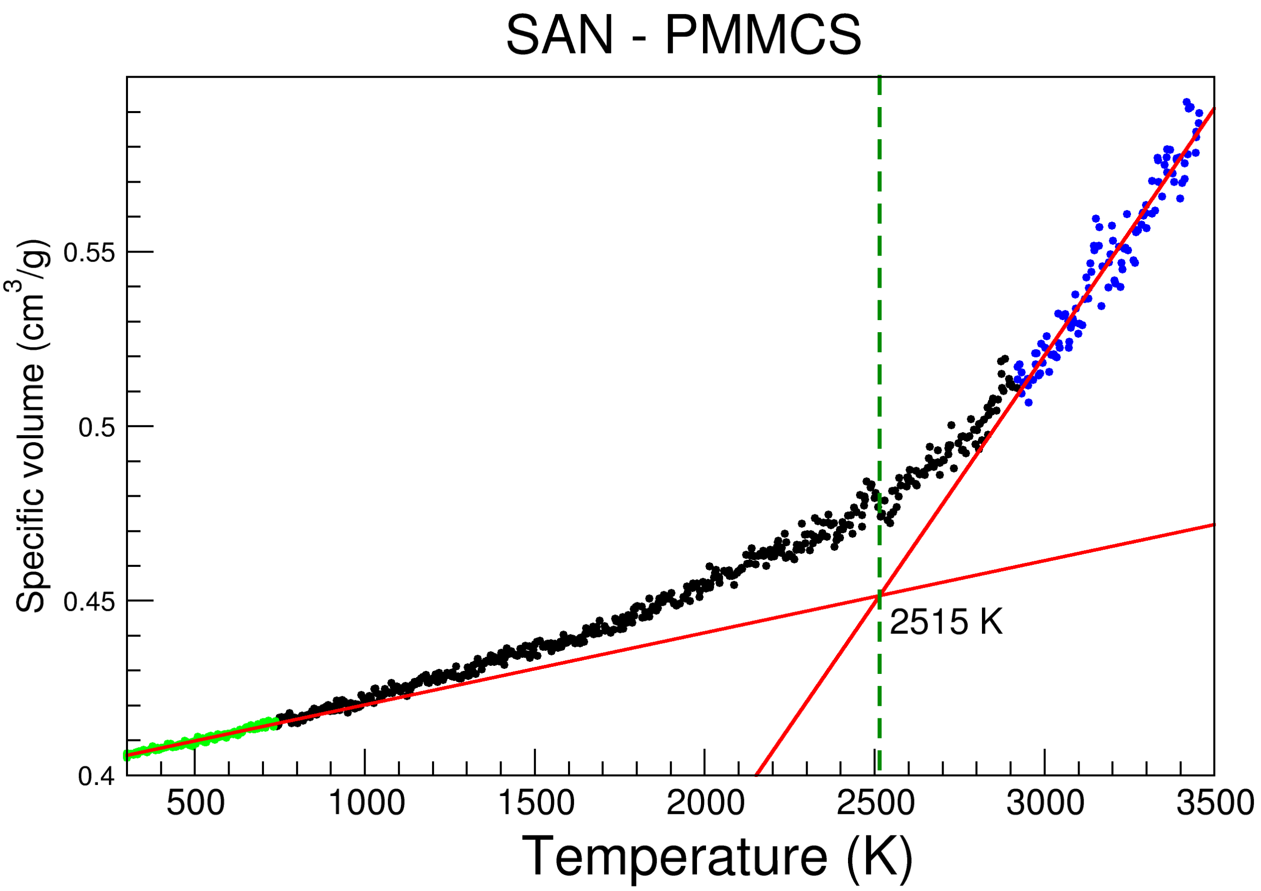

Figure S1. Schematic description of the method used to determine the glass transition temperature.

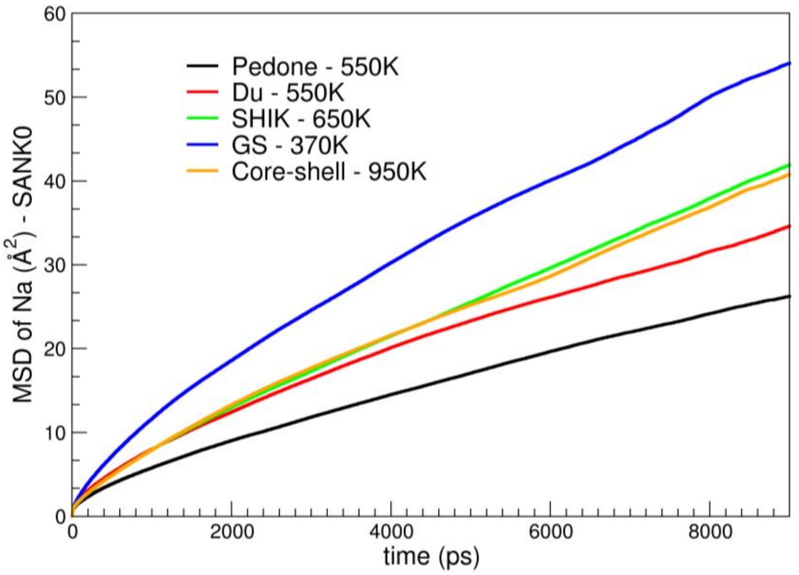

Figure S2. An example of mean square displacement for all potential. It is calculated for the glass SAN at the lower used temperature, as described in legend.

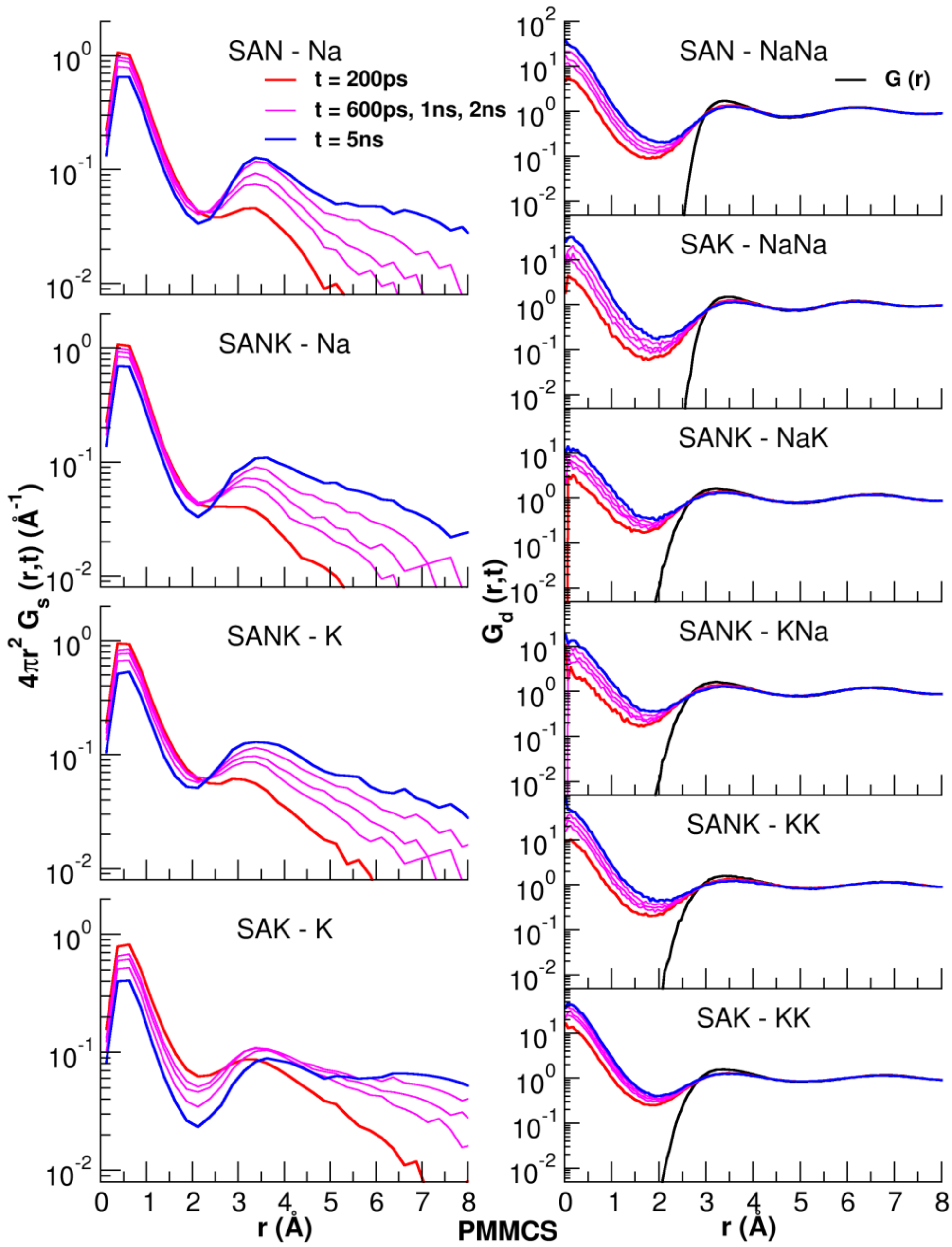

Figure S3. Self (left panels) and distinct (right panels) van Hove correlation functions at different simulation times for alkali ions in the three glasses simulated using the PMMCS potential.

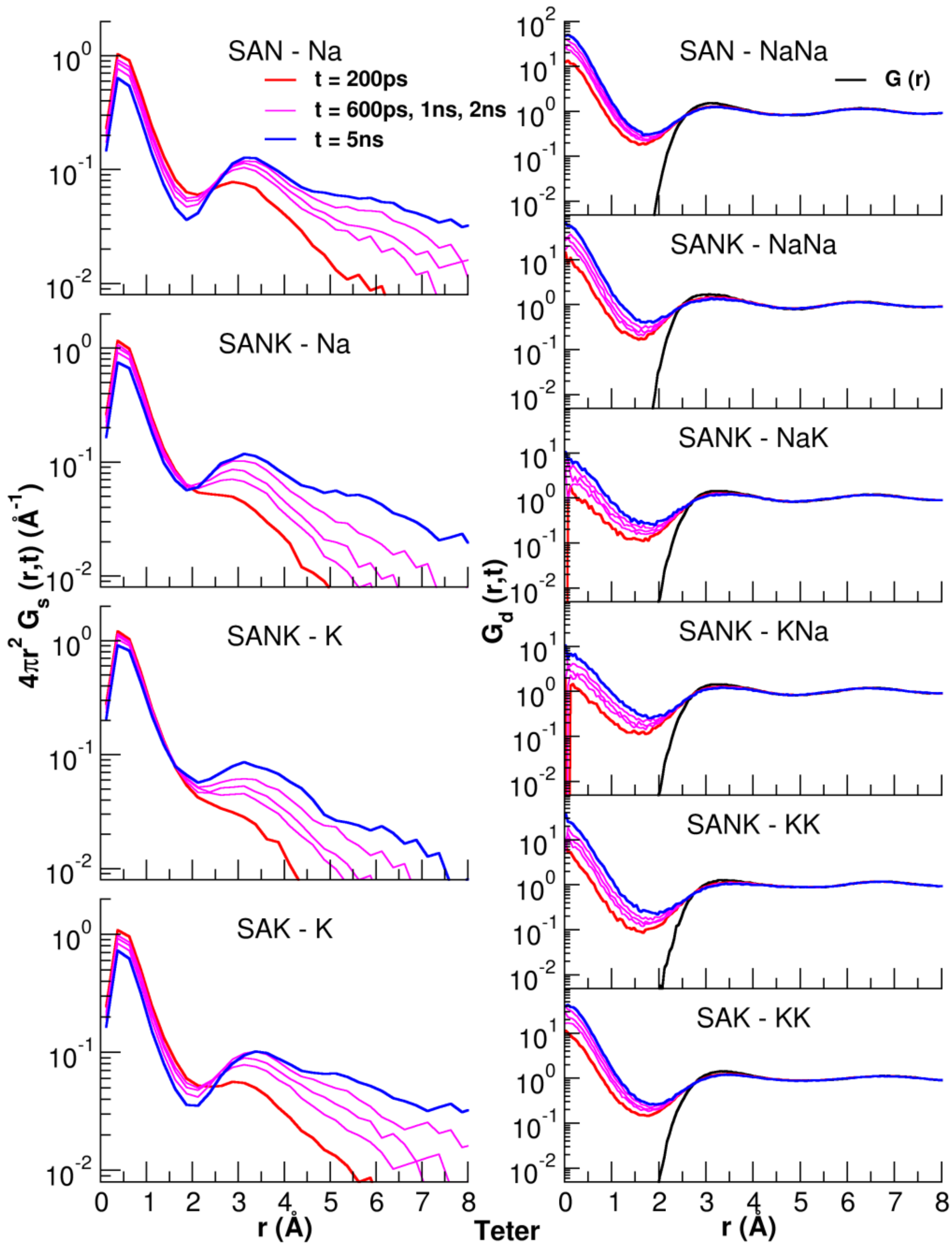

Figure S4. Self (left panels) and distinct (right panels) van Hove correlation functions at different simulation times for alkali ions in the three glasses simulated using the Teter potential.

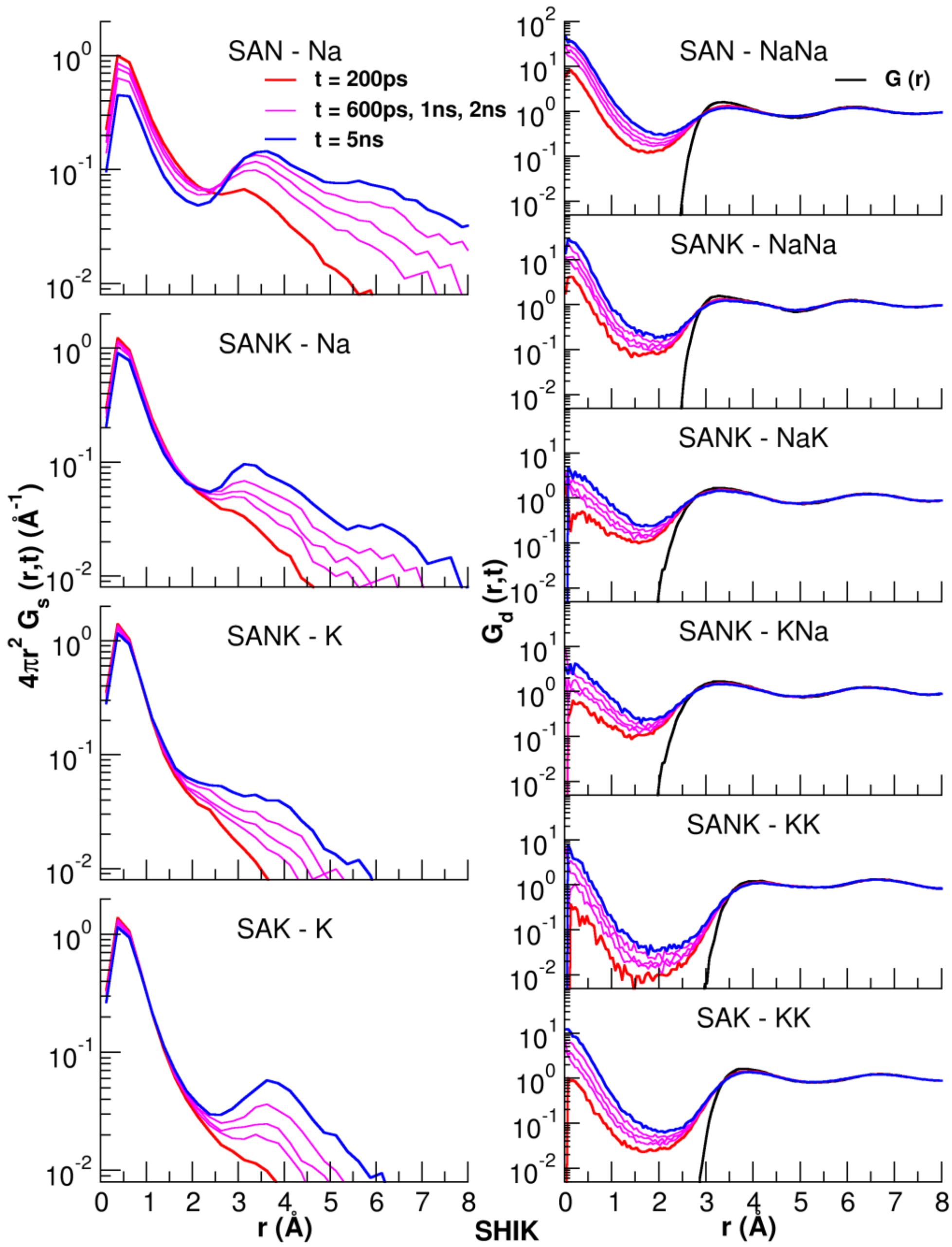

Figure S5. Self (left panels) and distinct (right panels) van Hove correlation functions at different simulation times for alkali ions in the three glasses simulated using the SHIK potential.

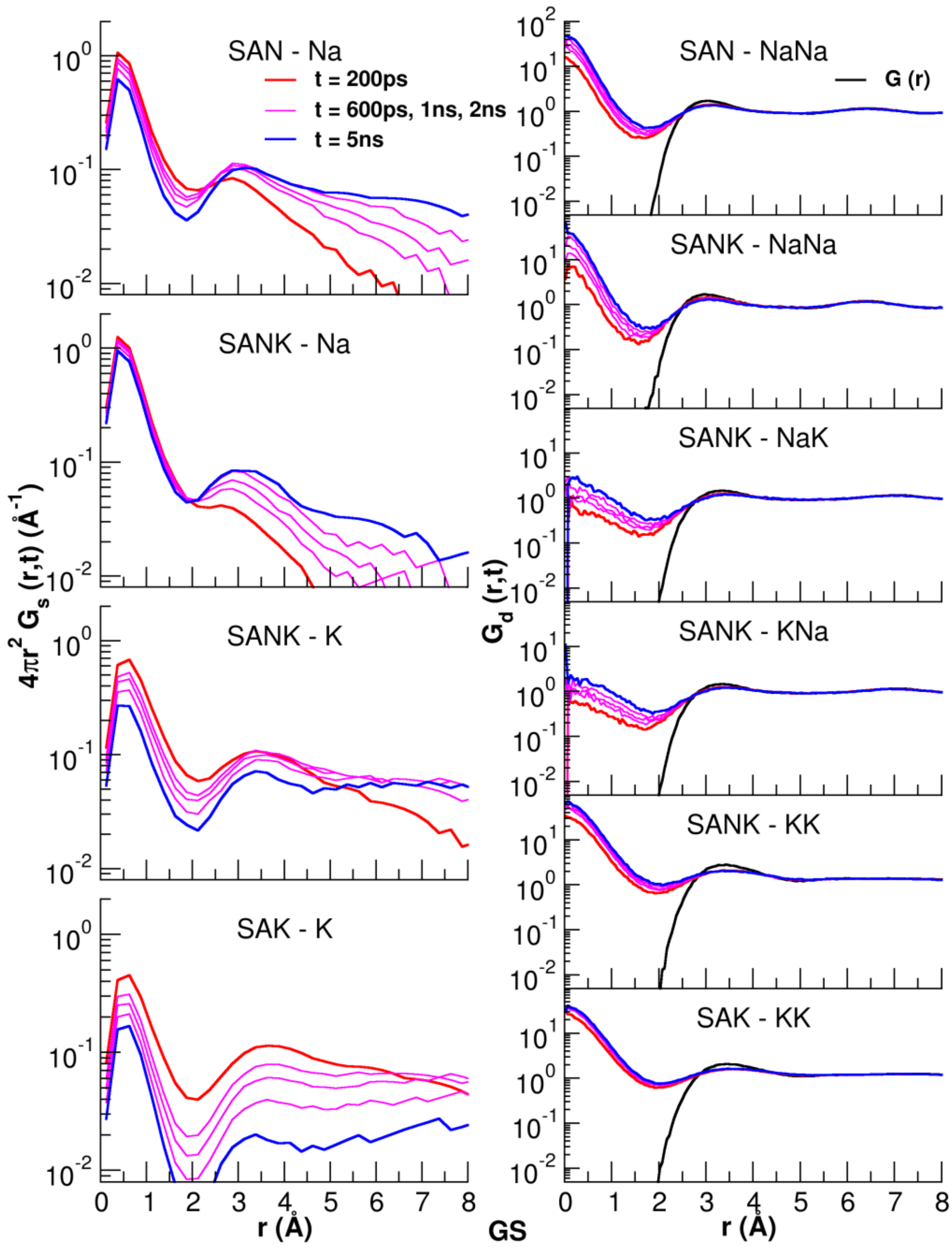

Figure S6. Self (left panels) and distinct (right panels) van Hove correlation functions at different simulation times for alkali ions in the three glasses simulated using the GS potential.

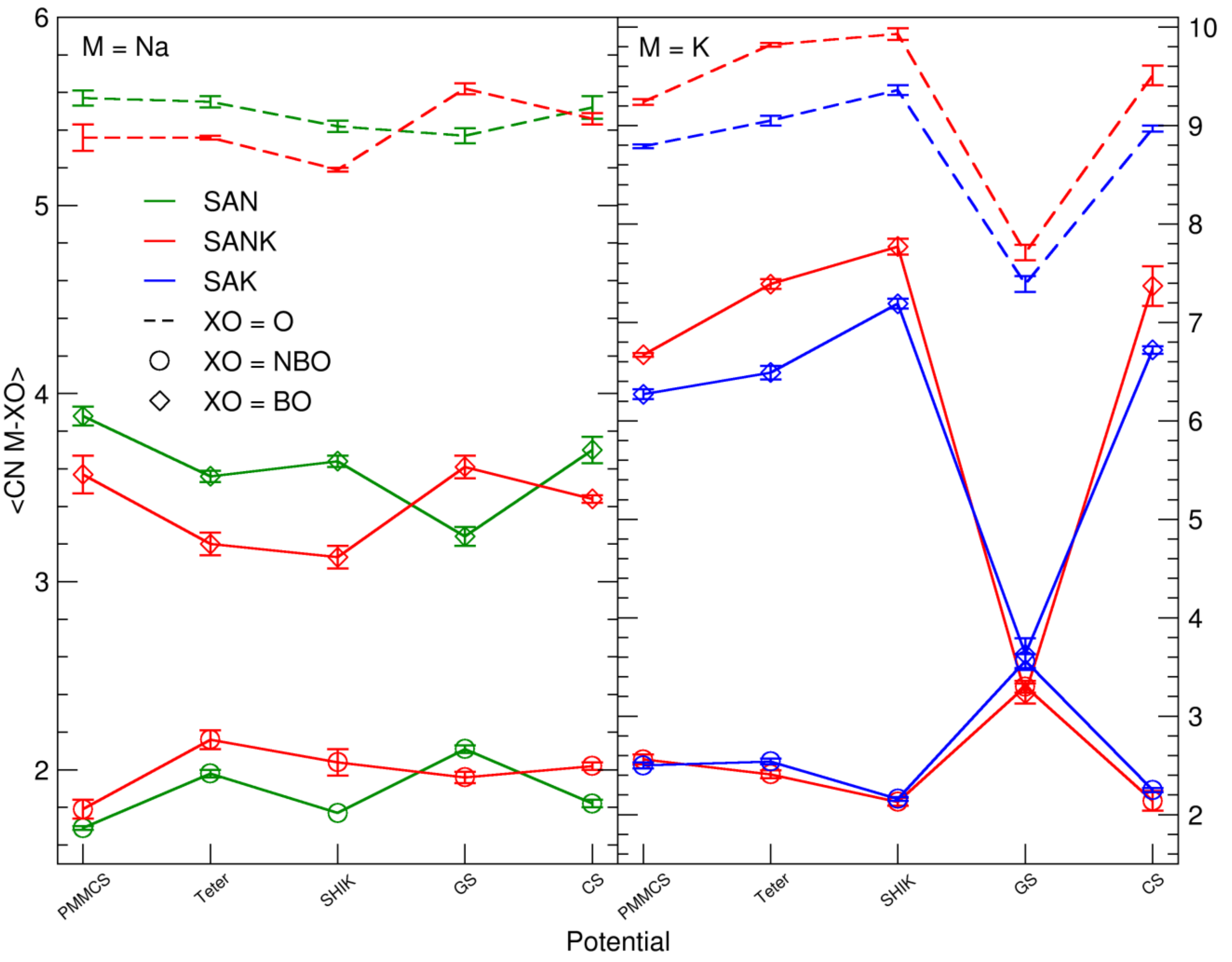

Figure S7. Average Na-O (left) and K-O (right) coordination numbers (and partitioning between BO and NBO species) of the three glasses studied as a function of the different interatomic potentials used.

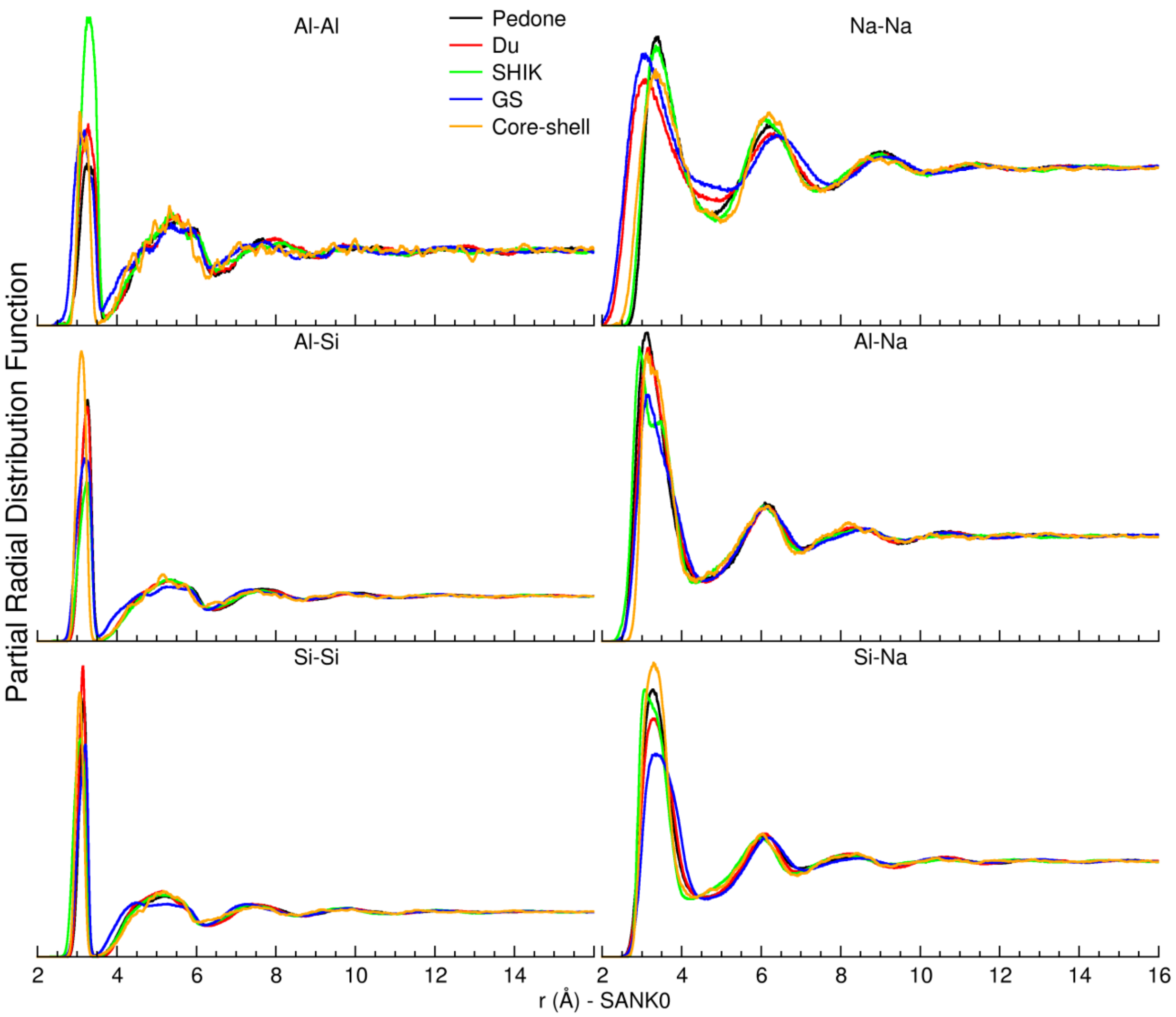

Figure S8. Cation-Cation pair distribution functions of the SAN glass computed using the different interatomic potentials.

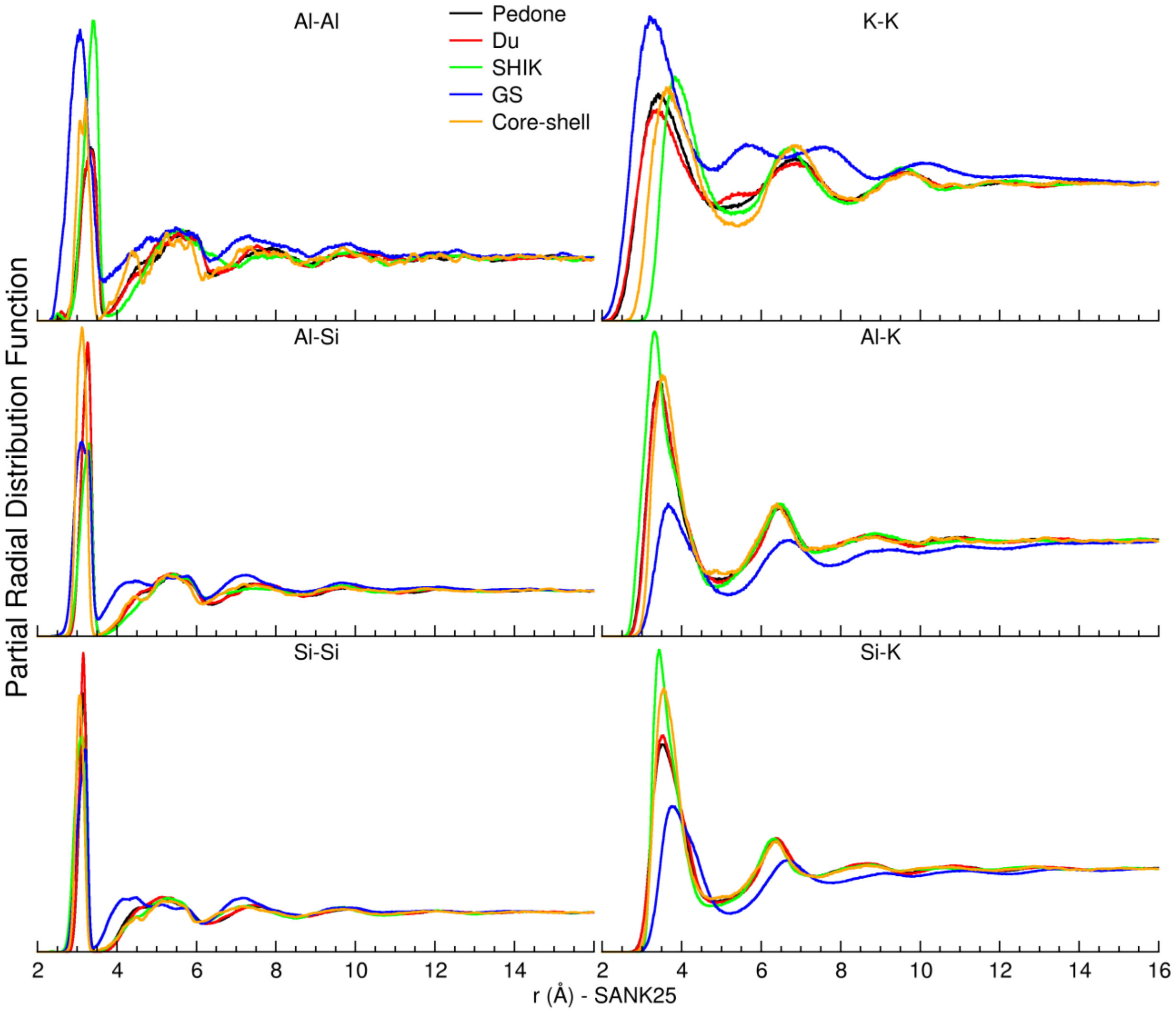

Figure S9. Cation-Cation pair distribution functions of the SANK glass computed using the different interatomic potentials.

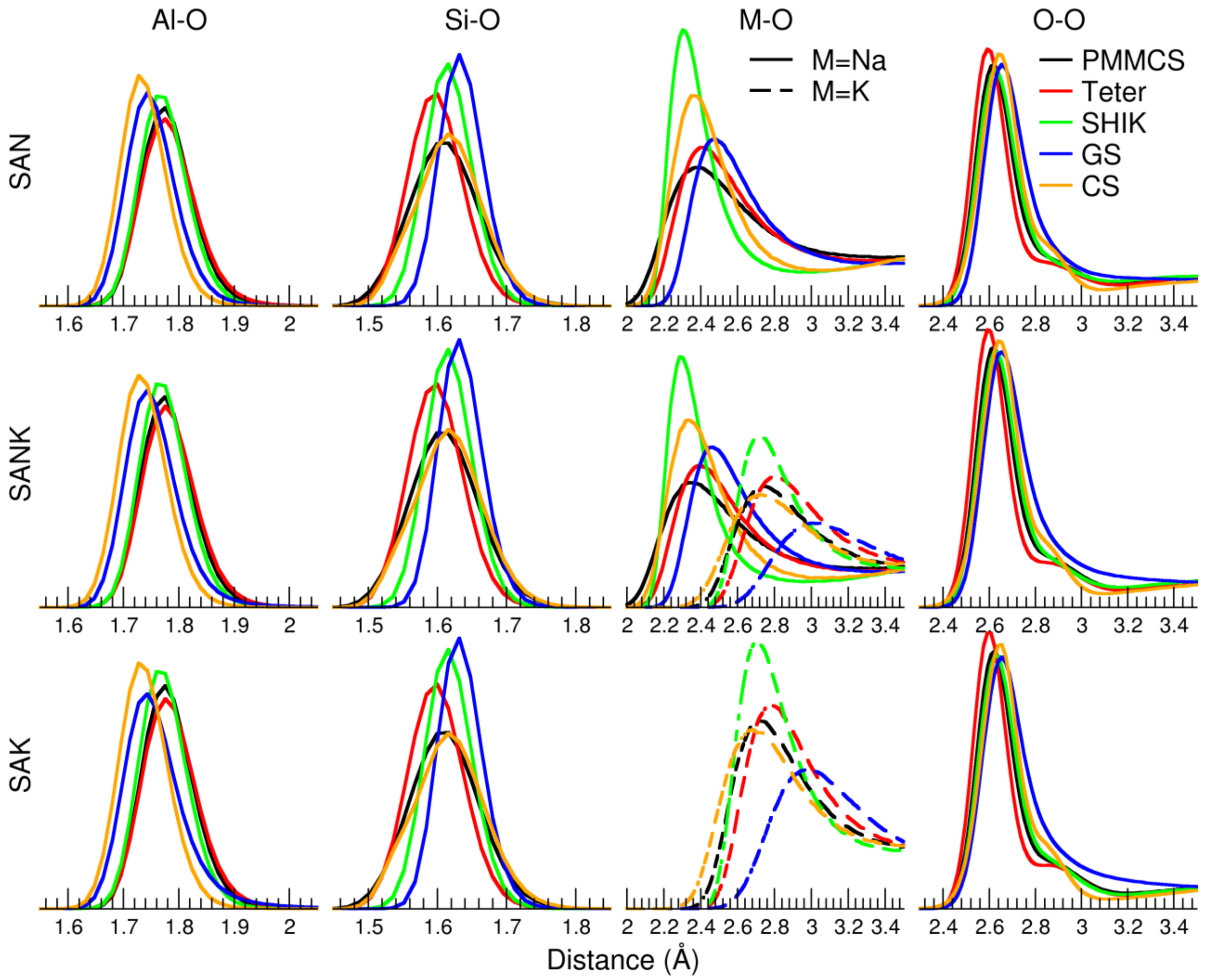

Figure S8. Si-O, Al-O, Na-O and O-O Pair Distribution Functions obtained with the different interatomic potential models for the investigated glass.

Analysis of the cations environments.

The Al-O, Si-O, Na-O, K-O and O-O PDFs obtained by means of the five interatomic potentials for the three investigated glasses are reported in **Figure S10,** whereas the numerical values of the most probable distances and the CNs are reported in **Table S8**. The Al-O bond distances range between 1.72 and 1.78 Å depending on the force-field used. The CS model provides the shorter distances followed by the GS (1.74 Å), SHIK (1.77 Å) and PMMCS and Teter potentials which provide the same results (1.78 Å) since they share the same potential for the Al-O interaction. It is interesting to note that the SHIK, Pedone and Du potentials are in better agreement with NEXAFS experiments on sodium aluminosilicate glasses that provide distances of 1.77 Å.[47]

Aluminium is mainly 4-fold coordinated but a small amount of 5-fold coordinated Al is usually found for PMMCS (<0.3%), Teter (<0.1%), SHIK (<0.3%) and GS potentials (<1.5%).

These trends are observed in all the glass compositions studied, denoting that the short-range order around Al is well defined and it is independent on the mixed alkali effect.

In the same manner, also the silicon environment is not affected by the glass composition. The Si-O PDFs show that, in this case, it is the Teter potential that provides the shorter distances (1.59 Å) followed by the PMMCS (~1.61 Å), CS and SHIK potentials (~1.62 Å) and by GS potential that provides the longer distances (~1.63 Å). All distances compare well with the EXAFS measurements of 1.61 Å.[53] It is worth to note that the SHIK and GS potentials present the more intense and narrower Si-O first peaks in the PDF, denoting a better-defined environment.

Regarding the Na-O and K-O environments, **Figure S10**, shows that in both cases the SHIK potentials provide PDFs with a more intense and narrower first Na/K-O peak centered at shorter distances with respect to the other interatomic potentials. As for the Na-O distances, the ordering is SHIK (2.29 Å) < PMMCS = CS (2.37 Å) < Teter (2.40 Å) < GS (2.47 Å), whereas for the K-O distances the values are 2.70-2.72 Å (SHIK, PMMCS and CS), 2.82 Å (Du) and 3.00-3.08 Å for the GS potentials.

The SHIK, Pedone and CS values are in better agreement with distances found in silicates (2.67 Å for K-O[54] and 2.30-2.36 Å for Na-O[53]) whereas the GS potentials compares better with the data reported for aluminosilicates (3.00-3.06 Å for K-O and 2.5-2.6 Å for Na-O)[47].

Figure S7 reports the average coordination numbers of sodium and potassium ions in the three glasses obtained using the different interatomic potentials. Sodium coordination ranges between 5.2-5.6 with the GS and CS potentials providing the larger values. PMMCS, Teter and SHIK potentials provides lower CN for sodium in the SANK glass, the opposite is observed with the GS potential and almost All the potentials show that the coordination of alkaline ions to oxygen is dominated by BO (3.2-3.8) rather than NBO (1.8-2.2). In general, PMMCS, Teter, SHIK and CS potentials provide similar Na-BO coordination numbers, whereas the GS potential gives smaller values for SANK and higher values for SAN.

Regarding potassium coordination, it is interesting to note that all the potentials provide similar total and partial coordination numbers with NBOs and BOs (K-O CN between 8.5-10 and K-BO CNs between 6.5-7.5) at the exception of the GS potential that produces lower total CNs (around 7.5) with an almost equal partition between BO and NBO. The NBO/BO ratio in the coordination sphere of sodium is between 0.5 and 0.67, whereas that of potassium is between 0.29 and 0.38 for all potentials but the GS one, for which it is equal to 1. This suggests that in general, the GS

potential a part, sodium has a more pronounced propensity to act as modifier with respect to potassium.

Finally, regarding the O-O distances the first peak of the PDF is centered at around 2.6-2.65 Å (**Figure S10**). In this case, the Teter potentials provides the shorter distances, whereas the GS potentials the longer ones.
